# Several steps of lateral gene transfer followed by events of ‘birth-and-death’ evolution shaped a fungal sorbicillinoid biosynthetic gene cluster

**DOI:** 10.1186/s12862-016-0834-6

**Published:** 2016-12-07

**Authors:** Irina S. Druzhinina, Eva M. Kubicek, Christian P. Kubicek

**Affiliations:** 1 Microbiology Group, Research Area Biochemical Technology, Institute of Chemical Engineering, TU Wien, Vienna, Austria; 2 Present address: Steinschötelgasse 7, 1100 Wien, Austria

**Keywords:** *Trichoderma*, *Penicillium*, Lateral gene transfer, Secondary metabolites, Birth-and-death evolution, PKS

## Abstract

**Background:**

Sorbicillinoids are a family of complex cyclic polyketides produced by only a small number of distantly related ascomycete fungi such as *Trichoderma* (Sordariomycetes) and *Penicillium* (Eurotiomycetes). In *T. reesei*, they are synthesized by a gene cluster consisting of eight genes including two polyketide synthases (PKS). To reconstruct the evolutionary origin of this gene cluster, we examined the occurrence of these eight genes in ascomycetes.

**Results:**

A cluster comprising at least six of them was only found in Hypocreales (*Acremonium chrysogenum, Ustilaginoidea virens*, *Trichoderma* species from section *Longibrachiatum*) and in *Penicillium rubens* (Eurotiales). In addition, *Colletotrichum graminicola* contained the two *pks* (*sor1* and *sor2*), but not the other *sor* genes. *A. chrysogenum* was the evolutionary eldest species in which *sor1, sor2, sor3, sor4* and *sor6* were present. *Sor5* was gained by lateral gene transfer (LGT) from *P. rubens*. In the younger Hypocreales (*U. virens, Trichoderma* spp.), the cluster evolved by vertical transfer, but *sor2* was lost and regained by LGT from *C. graminicola. SorB (=sor2)* and *sorD (=sor4)* were symplesiomorphic in *P. rubens,* whereas *sorA, sorC* and *sorF* were obtained by LGT from *A. chrysogenum*, and *sorE* by LGT from *Pestalotiopsis fici* (Xylariales). The sorbicillinoid gene cluster in *Trichoderma* section *Longibrachiatum* is under strong purifying selection. The *T. reesei sor* genes are expressed during fast vegetative growth, during antagonism of other fungi and regulated by the secondary metabolism regulator LAE1.

**Conclusions:**

Our findings pinpoint the evolution of the fungal sorbicillinoid biosynthesis gene cluster. The core cluster arose in early Hypocreales, and was complemented by LGT. During further speciation in the Hypocreales, it became subject to birth and death evolution in selected lineages. In *P. rubrens* (Eurotiales), two cluster genes were symplesiomorphic, and the whole cluster formed by LGT from at least two different fungal donors.

**Electronic supplementary material:**

The online version of this article (doi:10.1186/s12862-016-0834-6) contains supplementary material, which is available to authorized users.

## Background

Horizontal or lateral gene transfers (HGT and LGT) are important mechanisms of genome evolution that significantly contribute to the development of adaptive traits [1]. Although once considered a process of limited effect outside prokaryotes [2, 3], we now know that HGT and LGT have occurred in all major eukaryotic lineages (reviewed in [4]), including protozoans, plants, animals and fungi [5–7]. In fungi, HGT-driven gene innovation was shown to have resulted in refined repertoires of secreted and transporter proteins and increased metabolic capacities [8]. A survey of sixty fungal genomes detected hundreds of genes horizontally acquired from bacteria [9]. But the list of donors of fungal genetic material also includes plants [10], microbial eukaryotes [11, 12], and - most frequently - other fungi [13]. We will use the term LGT to describe the latter events.

Fungal secondary metabolites have a long history of positive (pharmaceuticals) and negative (toxins) impacts on mankind. Polyketides (PKS) make up a major group of them, most of which are formed by only a few, frequently not closely related species [14, 15]. The origin of PKS diversity has been explained as the result of gene duplication, HGT, LGT, recombination and domain shuffling [16]. However, most of these data have been obtained only for bacteria. Kroken et al. [17] postulated that the observed diversity in fungal PKS’s may not have resulted from HGT or LGT, but rather be due to birth-and-death evolution. However, increased sampling of genomic data from diverse taxonomic groups later provided evidence for the origin of several fungal PKS by HGT from bacteria, and also - in a few cases – by LGT from other fungi and plants [8, 18–22]. In almost all of these cases, translocation involved the whole secondary metabolite clusters (i.e. the PKS and the adjacently located genes encoding modifying enzymes, gene regulators and transporters) - rather than individual genes. To the best of our knowledge, the only exception is the demonstration of reacquisition of biotin prototrophy in *Saccharomyces cerevisiae* by stepwise HGT from bacterial donors [23].

*Trichoderma* is a genus of mycotrophic ascomycetes. Baker et al. [24] have recently compared the polyketide synthase (PKS) inventory of three *Trichoderma* species (*T. reesei*, *T. virens* and *T. atroviride*) and showed that two polyketide synthase encoding genes - *pks10*, *pks11* - were unique to *T. reesei. Pks10* and *pks11* are located head-to-head in the center of chromosome 5 [25] and were shown to be responsible for the synthesis of sorbicillinoids [26]. These are complex cyclic polyketides, some of which have been shown to exhibit cytostatic and neuroprotective effects [27]. Sorbicillinoids are produced by *T. reesei* ([28, 29]; named *T. longibrachiatum* by the authors), but also some other fungal species belonging to the Sordariomycetes (e.g. *Verticillium*, *Acremonium*, *Paecilomyces;* for review see [27]) and the Eurotiomycete *Penicillium notatum* [30]. In support of this, a putative sorbicillinoid synthesizing cluster similar to the *T. reesei* cluster, is present in *P. rubens* [26, 31]. Moreover, the *P. rubens* orthologue of *T. reesei pks11* (*pks13*) was shown to be essential for sorbicillinoid biosynthesis [32].

This limited, yet taxonomically widespread occurrence of sorbicillinoid biosynthesis in fungi led us to hypothesize that their evolution occurred by other mechanisms than vertical transfer. The goal of this study was to evaluate the evolutionary history of sorbicillin biosynthesis in *T. reesei* and other fungi. Here we show that this PKS cluster indeed originated from LGT, but in contrast to other reported cases [8, 18–22] it was not transferred as a whole cluster but formed by separate transfers of the individual genes from different donor species. The first almost complete cluster occurred in *A. chrysogenum,* from where it was transferred to *P. rubens*. In contrast, its further shaping in the Hypocreales occurred mainly via birth-and-death evolution and survived only in a few species including one of the most recent lineages of *Trichoderma,* the *Longibrachiatum* section.

## Results

### Identification of homologues of the sorbicillinoid biosynthetic clusters in Ascomycetes

To identify gene clusters potentially involved in sorbicillinoid biosynthesis in fungi, we first searched the National Center for Biotechnology Information (NCBI) protein database with the two PKS10- and PKS11- encoded proteins of *T. reesei*, which represent a non-reducing and a reducing PKS respectively, by bidirectional BLASTP (see Methods, (Additional file 1: Figure S1). Genes encoding proteins with highest similarity to both PKS10 and PKS11 were identified from the plant pathogenic fungus *Colletotrichum graminicola* (Sordariomycetes, Glomerellales)*,* the opportunistic cephalosporin C-producer *Acremonium chrysogenum* (Sordariomycetes, Hypocreales), the “rice false smut” causing pathogen *Ustilaginoidea virens* (Sordariomycetes, Hypocreales) and *Penicillium rubens* (Eurotiomycetes, Eurotiales)*.* Genes encoding proteins with still high similarity to PKS10 and PKS11 were also found in several other fungi (Eurotiomycetes and Sordariomycetes for PKS11, and – in addition – Dothidiomycetes for PKS10), but only the four species named above contained both of them.

Baker et al. [24] reported that PKS11 and PKS10 are unique to *T. reesei,* based on the absence in other *Trichoderma* species for which genome sequences were available at that date. Since the genomes of eight more *Trichoderma* spp. (i.e. *T. harzianum, T. asperellum, T. hamatum, T. gamsii, T. longibrachiatum, T. citrinoviride* and *T. parareesei*) are now available ([32–36]; http://genome.jgi.doe.gov/programs/fungi/index.jsf), we also screened them for the presence of *pks10* and *pks11* orthologs. The two genes were only found in *T. longibrachiatum, T. citrinoviride* and *T. parareesei,* which all are – as *T. reesei* – members of the *Longibrachiatum* Section of *Trichoderma* [37].

**Fig. 1:**
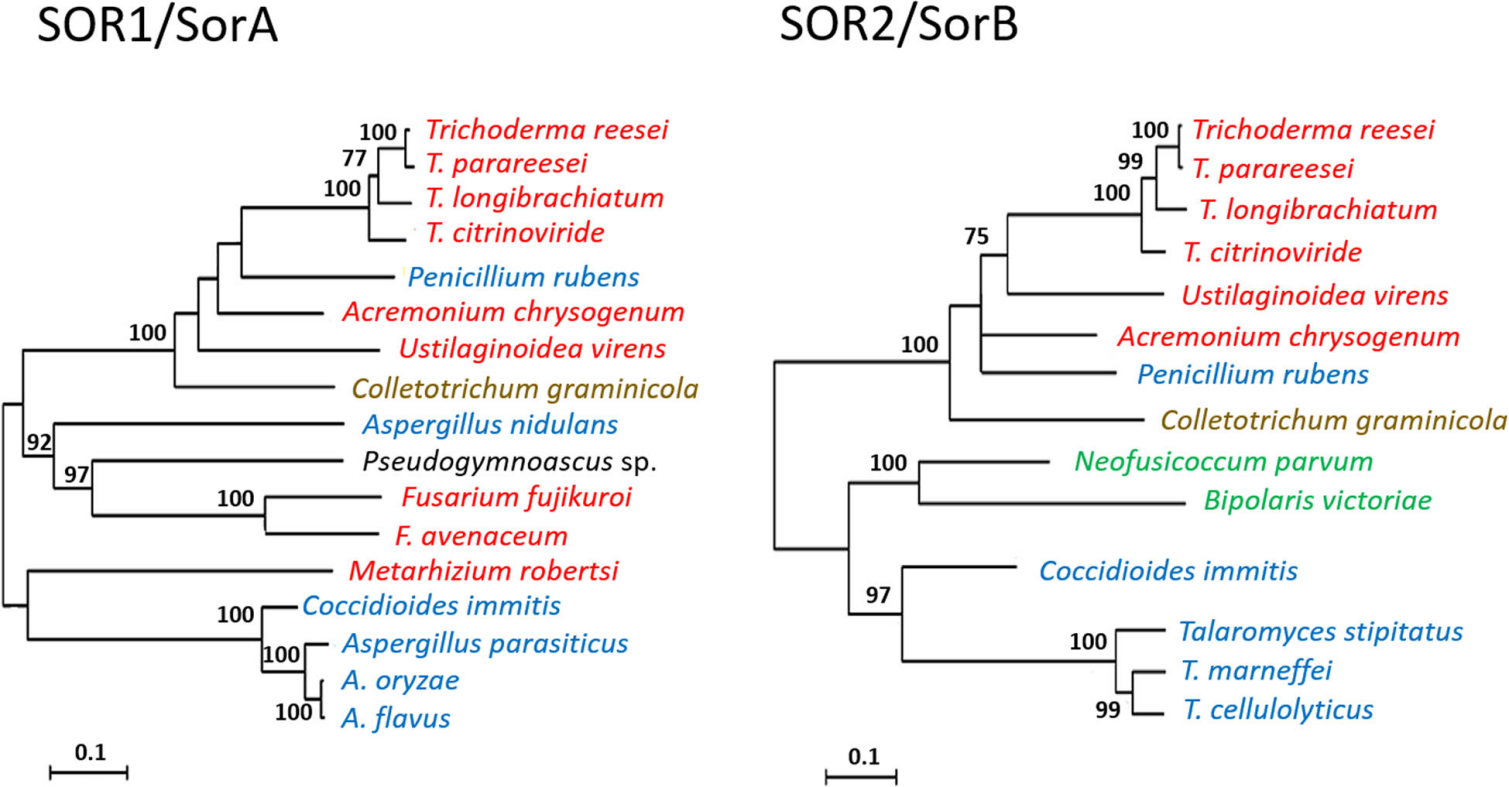
Phylogenetic analysis of SOR1/SorA (*T. reesei* PKS11) and SOR2/SorB (*T. reesei* PKS10) proteins by PhyML. Numbers at the nodes indicate the boostrap (1000 replica) support. Species printed in red belong to Hypocreales, brown to Glomerellales, black Pseudoeurotiaceae (all Sordariomycetes), blue Eurotiomycetes, green Dothidiomycetes. Accession numbers for proteins are given in Additional file 9: Table S1

The PKS10 and PKS11 orthologs that were retrieved by BLASTP and by screening of the *Trichoderma* genomes shows that they form a significantly supported clade that contained all those species in which genomes both PKSs were present. *C. graminicola* occurred at a basal position in this clade (Fig. 1). To indicate that these two genes are part of the sorbicillin biosynthetic cluster, we will – in agreement with [26] - further name them *sor1* (=*pks11*) and *sor2* (=*pks10*) throughout the manuscript.

**Fig. 2:**
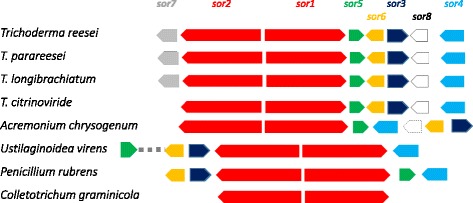
Architecture of the sorbicillinoid biosynthesis gene cluster in those fungi that possess SOR1/SorA and SOR2/SorB orthologues. Red: PKS, blue/dark blue: Zn2Cys6 transcription factor; green, FAD monooxygenase; orange, MSF transporter; black, short chain reductase/dehydrogenase; white, FAD-dependent oxidase. The dotted cluster in *A. chrysogenum* specifies an unknown protein. Accession numbers for all proteins shown are given in Additional file 9: Table S1

The sorbicillinoid biosynthetic gene clusters in *T. reesei* and *P. rubens* comprise 8 and 6 genes, located on chromosomes 5 and 1, respectively ([25, 26], http://www.ncbi.nlm.nih.gov/protein/CAP95405) (Fig. 2)*. sor3/sorC* and *sor4/sorD* encode binuclear Zn2Cys6 transcription factors, of which *sor4* is essential for the biosynthesis of sorbicillinoids in *T. reesei* [26]; *sor5/sorE* encodes a FAD-dependent monooxygenase responsible for the oxidative de-aromatisation of sorbicillin and dihydrosorbicillin to sorbicillinol and dihydrosorbicillinol, respectively [38]; and *sor6/sorF* encodes a transporter of the major facilitator superfamily (MFS).

We consequently analysed whether the other species that contain *sor1* and *sor2* would indeed also contain the other 4 or 6 genes that are present in the *P. rubens* and *T. reesei* cluster, respectively, and have them organized in a genomic cluster (Fig. 2): *C. graminicola* contained no homologues of any of them, but *A. chrysogenum, U. virens* and the other 3 *Trichoderma* spp. contained *sor3, sor4, sor5* and *sor6* (i.e. the genes encoding the two transcription factors*,* the MFS transporter the FAD-dependent monooxygenase, respectively). All of them were located in immediate vicinity of *sor1* and *sor2*, although *U. virens sor5* is located a few genes farther apart than in the other fungi.

The cluster in *T. reesei* contained two further genes – *sor7* and *sor8* - that were absent from most other fungi: *sor7*, encoding a short-chain dehydrogenase/reductase, for which a *P. rubens* ortholog (CAP92704.1) - is present in the genome but not located in the vicinity of the sorbicillinoid gene cluster In *A. chrysogenum,* another gene of unknown function is found at the position of *sor7. sor8* encodes an FAD-dependent oxidase (Fig. 2), of which an ortholog is present in *A. chrysogenum* and *U. virens*, but not located in the vicinity of the sorbicillinoid cluster, and absent from the *P. rubens* genome (Fig. 2).

We also looked at possible synteny of the 5′ and 3′ flanking regions of the cluster: while there was considerable synteny between the four *Trichoderma* spp., no synteny was found between *Trichoderma* and the other fungi possessing the sorbicillinoid biosynthesis cluster.

We tested the null hypothesis that the phylogenetic history of SOR3 – SOR8 was consistent with a vertical transfer within fungi by implementing phylogenetic analyses (Additional file 2: Figure S2 A-F). This shows that only SOR5 forms a strongly supported clade containing – except for *C. graminicola* - all species that also contain the SOR1 and SOR2 proteins. SOR3, SOR4 and SOR6 are distributed in several clades, which – even after collapsing branches with poor bootstrap support (<75%) are not concordant with the established Ascomycota phylogeny (cf. [39]). On the other hand, SOR7 and SOR8 display a phylogeny that strongly resembles the Ascomycota phylogeny (see below; cf. Additional file 3: Figure S3). They are also present in *Trichoderma* spp. which lack the sorbicillinoid biosynthetic cluster.

### Evolution of the sorbicillinoid gene cluster in filamentous fungi

The above described discordance between the phylogeny of SOR1-SOR6 homologues and the Ascomycota phylogeny suggested that they may have arisen by LGT from different ancestors. To test this hypothesis, we applied three complementary approaches: the bipartition dissimilarity test implemented in T-REX [40], which identifies HGT/LGT events by quantifying the proximity between two phylogenetic trees using a refinement of the Robinson and Foulds distance [41, 42]; the reconciliation of each gene tree to the fungal species phylogeny, thereby assigning costs to gene duplications, HGT/LGT, gene loss, and incomplete lineage sorting, as implemented in Notung [43]; and the Jane software tool that uses a polynomial time dynamic programming algorithm in conjunction with a genetic algorithm to find solutions pairs of trees [44]. We accept proof for HGT/LGT only for those cases where (i) at least two of these programs provided consistent results that were not rejected by the third, and (ii) where the protein tree topology was contradictory to the Ascomycota phylogeny and could not be more parsimoniously reconciled using a combination of differential gene duplications (GD) and gene loss.

The evolution displayed by the results from this analysis (Additional file 4: Figure S4, Additional file 5: Table S2) are summarized in Table 1: evidence for LGT was obtained for *A. chrysogenum* (SOR4), *Trichoderma* and *U. virens* (SOR2, SOR3), and *P. rubens* (SorA, SorC, SorE and SorF). Interestingly, three of the genes of *P. rubens* (SorA, SorC and SorF) were obtained from *A. chrysogenum*, whereas SOR4 of *A. chrysogenum* was obtained from *P. rubens* (SorD), indicating frequent LGTs between these two species. In *Trichoderma* and *U. virens*, only SOR2 appears to have been obtained by LGT from *C. graminicola*.

**Table 1:**
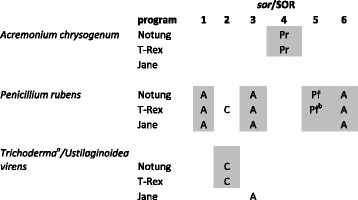
Summary of LGT events detected by T-Rex, Notung and Jane

Approved LGTs are highligthened by a grey background

*Abbreviations*: *A. chrysogenum, C. graminicola*, Pf* P. fici*, Pr *P. rubens*

^a^only sect. *Longibrachiatum*

^b^transfer to an ancestor of *P. rubens*

With the exception of SOR2, neither *U. virens* nor the four *Trichoderma* spp. appear to have received any of the other cluster genes by LGT. No LTG events could be inferred for *sor7* and *sor8* (neither by Notung, T-REX nor Jane) which is in agreement with the observation that these genes occupied positions concordant with Ascomycota phylogeny (*vide supra*).

### *Trichoderma* SOR1 and SOR2 evolved by purifying selection

The *Longibrachiatum* Section of *Trichoderma* is one of the most recent branches in *Trichoderma* evolution [45]. The fact that we were unable to identify the *sor* genes in less evolutionary derived species of the genus but could not verify LGT as the mechanism of origin of the sorbicillioid gene cluster in the *Longibrachiatum* clade was thus unexpected. The alternative hypothesis to explain the absence of these genes in other species is that these genes have been lost. To test this hypothesis, we reconstructed the evolution of the eight SOR proteins by Count [46] and Gloome [47]. The results provided consistent evidence for loss of the respective genes in other infrageneric clades of *Trichoderma* and in those Hypocreaceae species that are close to *Trichoderma* but also lack them (Additional file 6: Figure S5). Interestingly, the ratio of the pairwise amino acid differences between SOR1/SorA and SOR2/SorB and the housekeeping genes used to construct the Ascomycota tree (see Methods), was significantly higher in the four *Trichoderma* spp. than in *U. virens, P. rubens* or *A. chrysogenum* (Table 2). This would be typical for LGT, as was found for SOR2. However, since *sor1* has not been obtained by LGT, it may as well be due to a higher rate of evolution of these two genes in *Trichoderma* section *Longibrachiatum*. Determination of the K_a_/K_s_ ratio for the *Trichoderma sor1* and *sor2* genes yielded values around 0.1, suggesting the operation of strong purifying selection.

**Table 2:** Pairwise amino acid distance of the four housekeeping genes (HKG). SOR1 and SOR2 between *Trichoderma citrinoviride* and other fungi

	HKG	SOR1	SOR2
*Trichoderma reesei*	0.016	0.084	0.088
*Trichoderma parareesei*	0.025	0.095	0.09
*Trichoderma longibrachiatum*	0.009	0.1	0.095
*Acremonium chyrsogenum*	0.21	0.314	0.377
*Visoclavia virens*	0.252	0.373	0.397
*Colletotrichum graminicola*	0.35	0.386	0.476

### Sorbicillinoid cluster gene expression in *T. reesei*

**Fig. 3:**
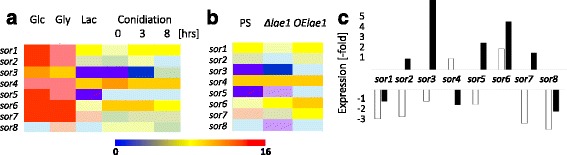
Heat plot of the expression of *sor1 - sor8* in *T. reesei* during **a** growth on different carbon sources and onset of sporulation; Glc - glucose; Gly - glycerol, Lac - lactose, **b** during cultivation of a *lae1* knock-out and a *lae1* overexpressing strain on lactose, and **c** plotting of the ratio between the parent strain to the *lae1* knock-out (*white bars*) and the *lae1* overexpressing strain (*black bars*), respectively

Many PKS synthesizing clusters in fungi are silenced [48]. We therefore used available oligonucleotide microarray data of *T. reesei* growing on glucose, glycerol, lactose or cellulose as carbon sources in submerged culture, or on glucose on agar plates to test whether the *sor* genes are indeed expressed. In fact the eight *sor* genes in the *Trichoderma* cluster are expressed at high levels under conditions of rapid growth (glucose, glycerol), whereas lower expression was detected on lactose which allows only slow vegetative growth (Fig. 3a). Most *sor* genes had only a low level of expression during asexual sporulation (Fig. 3a).

The protein methyltransferase LaeA is a major regulator of secondary metabolism in Eurotiomycetes and some Sordariomycetes [48]. Its *T. reesei* orthologue LAE1 regulates some but not all PKS genes [49]. As shown in Fig. 3c, a *lae1* knock-out mutant shows significantly decreased expression of *sor1* and *sor2*, and interestingly also of *sor7* and *sor8*. No increased expression was observed for these four genes in a strain overexpressing *lae1* under a constitutive promoter. However, the genes encoding one of the two transcription factors (*sor3*), the MFS transporter (*sor6*) and the FAD monooxygenase (*sor5*) were significantly upregulated by *lae1* overexpression.

## Discussion

Although HGT and LGT occur in the majority of cases by transfer of single genes only (for review see [9, 50], the transfer of multiple genes or gene clusters has also been shown [11, 19, 50–52], particularly for genes encoding proteins for secondary metabolite synthesis [18, 20, 53–59]. In contrast, our data show that the fungal sorbicillin biosynthesis cluster evolved by complementing symplesiomorphous genes by LGT from other fungal donors, which in *P. rubens* occurred in at least two steps. Based on the species phylogeny and the LGT events found, *A. chrysogenum* is the most ancient known taxon that contains an almost complete cluster that misses only the transcription factor *sor4*, and which it obtained by LGT from *P. rubens*. The more recent Hypocreales (*U. virens* and the four *Trichoderma* spp. of section *Longibrachiatum*) regained one of the PKSs (*sor2*) from *C. graminicola*, implying that this gene was lost in one of the Hypocreales that are more recent than *A. chrysogenum*.

In contrast, the cluster is missing in the Eurotiales with the exception of *P. rubens*, at least with respect to species whose genome sequence is available. The Eurotiales only contain orthologs of the PKS SorB and the transcription factor SorD, which lends to speculate that these two genes are involved in the synthesis of another polyketide. It is interesting to note that three missing genes (*sorA, sorC* and *sorF*) were obtained from *A. chrysogenum*, to which *P. rubens* transferred its *sorD*, indicating a history of a frequent gene exchange between these two fungi*.* We cannot say, however, whether the LGT from *A. chrysogenum* to *P. rubens* occurred in one or several steps. It is also interesting that *sorE* (encoding the FAD monooxygenase crucial for sorbicillin formation [38]) had not been transferred to *P. rubens* but to an unknown Eurotiales ancestor from *P. fici.* Thus *sorE* must have been present in *P. rubens* before LGT of *sorA, sorC* and *sorF*.

The absence of *sor3 – sor6* from *C. graminicola* could be due to gene loss. An alternative hypothesis, however, would be that SOR1 and SOR2 are synthesizing a different polyketide than sorbicillin in this fungus. Indeed, an annotation of the genes flanking the *C. graminicola sor1*/*sor2* locus revealed an adjacent oxidoreductase gene whose encoded protein exhibited 83% amino acid similarity to an oxidoreductase CtnB involved in citrinin biosynthesis in *Monascus aurantiacus* (Eurotiales) [60], and a putative aldehyde dehydrogenase (Additional file 7: Figure S6). We therefore assume that the resulting polyketide synthesized by *Colletotrichum* is (or was) probably not a sorbicillinoid. Sorbicillinoid were thus in fact first produced in *A. chrysogenum* or closely related but as yet unknown ancestor.

Despite of the occurrence of the sorbicillinoid gene cluster in only *U. virens* and *Trichoderma* spp. from section *Longibrachiatum*, we found (with the exception of *sor2*) no evidence for their origin by further LGT events. Instead, our data show that the cluster evolved by vertical transfer, and has been lost by the operation of massive birth-and-death evolution [61] within the Hypocreales. In fact, a scenario of gene duplications followed by gene loss has earlier been suggested for the evolution of fungal non-reducing polyketide synthases [17], and claimed to be responsible for the todays patchy distribution of distantly related secondary metabolites.

Yet our finding that the sorbicillinoid cluster only survived in *Trichoderma* species belonging to section *Longibrachiatum* is interesting. It is consistent with the formation of the characteristic yellow pigment secreted by these species [62], because sorbicillinoids have a characteristic yellow-orange color [63]. Species from this section are known to have smaller genomes than other *Trichoderma* spp. and represent one of the youngest phylogenetic clades of the genus [38, 45]. The fact that the sorbicillinoid gene cluster has been maintained in these species but not in other *Trichoderma* spp. suggests that the respective products are of selective importance to fungi from this section. This is also supported by our findings of strong purifying selection acting on *sor1* and *sor2*. Unfortunately, the function of sorbicillinoids is not known yet: although some sorbicillinoids were reported to inhibit the growth of tumour cells [27, 64], they usually display only low inhibitory activity against bacteria and fungi [27]. Their role as components of antagonism against other organisms is unlikely. Rather, their ecological importance may reside in their high antioxidant and radical scavenging activity [27, 65]. Our findings of high expression of the *sor* genes in *T. reesei* under conditions of fast growth, but not during sporulation, supports a role of sorbicillinoids in vegetative growth, which is corroborated by finding them in high concentrations during submerged growth of *T. reesei* (Additional file 8: Table S3). Interestingly, *sor1* and *sor2* are also strongly upregulated upon confrontation of *T. reesei* with plant pathogenic *Thanatephorus* spp./*Rhizoctonia solani* (Cantharellales, Basidiomycota) [66]. At a first glance, this contradicts the above conclusion that sorbicillinoids are not involved in antagonism. However, the protection against radicals formed by reactive oxygen species is an important defence reaction of fungi, plants and higher eukaryotes when confronted by other organisms [67–70]. It will be intriguing to find out whether the sorbicillinoids indeed play such a role and - if so – why their biosynthesis was just maintained in only a small group of fungal species.

## Conclusions

Tracking the evolution of secondary metabolite synthesizing gene clusters by LGT or HGT have so far in most cases been restricted to the detection of transfer of the whole clusters between two fungi. Our findings show how a fungal secondary metabolite cluster was assembled by individual genes from different fungi by LGT before it became subject to birth-and-death evolution in selected lineages.

## Methods

### Identification of sorbicillinoid biosynthetic genes in fungi

The eight proteins of the sorbicillinoid biosynthetic cluster in *T. reesei* were used in a preliminary sequence similarity search by BLASTP of the NCBI database. One hundred best hits were collected. In addition, we searched the genomes of *T. longibrachiatum, T. citrinoviride, T. asperellum, T. hamatum* and *T. parareesei* for homologues to SOR1 - SOR8. Since the latter two are not available in a public database, we prepared a local BLAST databases for these two fungi. All these sequences were then aligned by CLUSTALW [71], and subjected to phylogenetic analysis with PhyML 3.0 using the Dayhoff model and 1000 boostrap replica [72]. The topology of the resulting tree was analysed, and all proteins that formed clades not related to that comprising the *T. reesei* proteins were removed. The resulting collection of sequences was re-aligned with MUSCLE [73] and CLUSTALW [71] and edited by GBLOCKS [74] to identify potential differences in the phylogenetic reconstruction due to the use of different methods. Individual trees were reconstructed with the individual edited protein alignments with PhyML 3.0 using 1000 bootstrap repetitions, and their topology concordance confirmed. The four alignments were then concatenated, and Bayesian analysis performed with TOPALI v2.5 [75], using the WAG model, gamma substitution and 100,000 generations.

### Ascomycota tree reconstruction

To reconstruct the reference Ascomycota phylogeny containing all fungi putatively involved in the LGT events described in this paper, the amino acids inferred from four nuclear genes, which were shown to be good phylogenetic markers for fungal species trees reconstruction (i.e. histone acetyltransferase subunit of the RNA polymerase II holoenzyme, FG533; NAD-dependent glutamate dehydrogenase, FG570; translation initiation factor eIF-5, FG832; and Tsr1p, a protein required for processing of 20S pre-rRNA, MS277) were retrieved from FunyBase [76] (http://genome.jouy.inra.fr/funybase). Proteins from species not contained in FunyBase were retrieved by BLASTP search of the GenBank (http://www.ncbi.nlm.nih.gov/genbank/), the Joint Genome Institute (http://genome.jgi-psf.org/programs/fungi/index.jsf?projectList), EnsemblFungi (http://fungi.ensembl.org/index.html) and Broad Institute (http://www.broadinstitute.org/) databases (all databases accessed 28-12-2015). Their alignment, and analysis by PhyML 3.0 and Bayesian analysis were essentially performed as described above.

### Inferring HGT/LGT events

To test for the occurrence of HGT, three approaches were used: first, the bipartition dissimilarity test implemented in T-REX [40], which quantifies the proximity between two phylogenetic trees using a refinement of the Robinson and Foulds (RF) distance, was used by applying midpoint rooting and HGT identification by iteration. Second, a gene tree-species phylogeny reconciliation was performed in Notung, using its duplication, transfer, loss and ILS aware parsimony-based algorithm [43]. To this end, gene tree nodes with less than 0.90 SH-like local support were collapsed, and the resulting tree rooted and its polytomies resolved against the bifurcating species phylogeny. This resolved gene tree was then reconciled to the multifurcating, consensus species phylogeny using a duplication cost of 1.5, loss cost of 1 and ILS cost of 0, and the option to prune taxa not present in the gene tree enabled. Third, Jane version 4, a software tool for cophylogeny reconstruction problems that attributes costs to cospeciation, duplication, host switch, and sorting was used [44]. For our analyses we employed default cost settings, and the population size was set 50-fold the number of generations.

### Gene gain and loss analysis

Gene gain and loss was tested by two methods: (i) Count [46], which can perform ancestral genome reconstruction by posterior probabilities in a phylogenetic birth-and-death model. Rates were optimized using a gain–loss–duplication model, with default parameters and allowing different gain–loss and duplication–loss rates for different branches, and one hundred rounds of optimization. (ii) Gloome [47], which enables accurate inference of gain and loss events by a stochastic mapping approach, using a variable gain and loss ratio.

### Analysis of selection pressure by Ka/Ks ratio

Tajima’s D statistic [77] was determined with DNASp 5.0 [78], using a sliding-window approach.

### Transcriptome analysis

We used transcriptome data from our own earlier studies. These included: cultivation of *T. reesei* QM 9414 on D-glucose, glycerol, lactose, and wheat straw in batch cultures [79, 80], during induction of asexual sporulation [81], at the onset of confrontation with the basidiomycete *Thanatephorus spp./Rhizoctonia solani* [82], and during growth on lactose in *lae1* knock-out and *lae1*-overexpressing strains [51]. All transcriptome data were obtained by oligonucleotide array hybridization, using a high-density oligonucleotide microarray (Roche-NimbleGen, Inc., Madison, WI) with 60-mer probes representing the 9123 genes of *T. reesei*. Values were normalized by quantile normalization [83] and the RMA algorithm [84]. After elimination of transcripts that exhibited an SD >20% of the mean value within replicates, false discovery rates [85] were used to assess the significance of values. All transcriptome data and the related protocols are available at the GEO web site (http://www.ncbi.nlm.nih.gov/geo) under the accession numbers given in the cited papers.

## Additional files

Additional file 1: Figure S1. Architecture of Trire2: 73618 and Trire2:73621. The bar specifies the size of the proteins (in amino acid residues). Abbreviations: PKS, polyketide synthase; AT, acyltransferase; DH, dehydrogenase; AM, adenosyl-methionine transferase; ER, enoyl reductase; KR, keto reductase; TR, thioester reductase. (PDF 165 kb)
Additional file 2: Figure S2. Phylogenetic analysis of SOR3/SorC (A), SOR4/SorD (B), SOR5/SorE (C), SOR6/SorF (D), SOR7/SorG (E) and SOR8 (F) proteins by PhyML. Numbers at the nodes indicate the boostrap (1000 replicas) support. Numbers at the nodes indicate the bootstrap (1000 replicas) support. Colour codes are used as in Fig. 1. In addition, bright brown specifies members of the Sordariales. Accession numbers for all proteins shown are given in Additional file 9: Table S1. (PDF 368 kb)
Additional file 3: Figure S3. PhyML evolutionary tree of fungi, using protein sequences of the histone acetyltransferase subunit of RNA polymerase II, NAD-dependent glutamate dehydrogenase, translation initiation factor eIF-5, and Tsr1p, a protein required for processing of 20S pre-rRNA. For further details, see Methods. Species that contain a sorbicillinoid biosynthesis cluster are given in red. Donor species are printed in bold. Of *Trichoderma*, only *T. reesei* is shown for simplicity. (PDF 297 kb)
Additional file 4: Figure S4. Output trees of the analysis of SOR1-SOR8 by Notung (a), T-Rex (b) and Jane (c). In (a), yellow arrows indicate LGT, red D indicate duplication events; in (b), species names are abbreviated due to constraints of the program, but can easily be identified by comparing them to the species shown in (a) and (c); also note that of *Trichoderma* sect. *Longibrachiatum*, only *T. reesei* was used in these analyses; in (c), black lines identify the species tree, whereas blue lines indicate the protein tree. Lines with arrows show LGT, accompanied by support values. (PDF 673 kb)
Additional file 5: Table S2. Statistics of T-Rex, Notung and Jane analyses. (DOCX 15 kb)
Additional file 6: Figure S5. Gain and loss of SOR1 – SOR6 in the Hypocreales. Open bars indicate gene loss, number at the nodes indicate the respective loss rates. (PDF 130 kb)
Additional file 7: Figure S6. Gene structure of the 3′ end of supercontig 46 of the *C. graminicola* genome sequence (http://genome.jgi.doe.gov/Colgr1/Colgr1.home.html). No further genes are located 3′ of Colgr1:8017. (PDF 219 kb)
Additional file 8: Table S3. Trichodermol concentration in the culture fluid of *T. reesei*. (DOCX 12 kb)
Additional file 9: Table S1. Accession numbers of proteins shown in Figs.1, 2 and 3. (DOCX 32 kb)

## Abbreviations

GD: Gene duplications
HGT: Horizontal gene transfer
LGT: Lateral gene transfer
MFS: Major facilitator superfamily
PKS: Polyketide synthase
