# Supplementary figures and images for: Several steps of lateral gene transfer followed by events of ‘birth-and-death’ evolution shaped a fungal sorbicillinoid biosynthetic gene cluster
Source: BMC Evol Biol. 2016 Dec 7;16:269. doi: 10.1186/s12862-016-0834-6 (PMC5182515; doi:10.1186/s12862-016-0834-6)

Additional file 1: Figure S1

SOR1 = PKS11

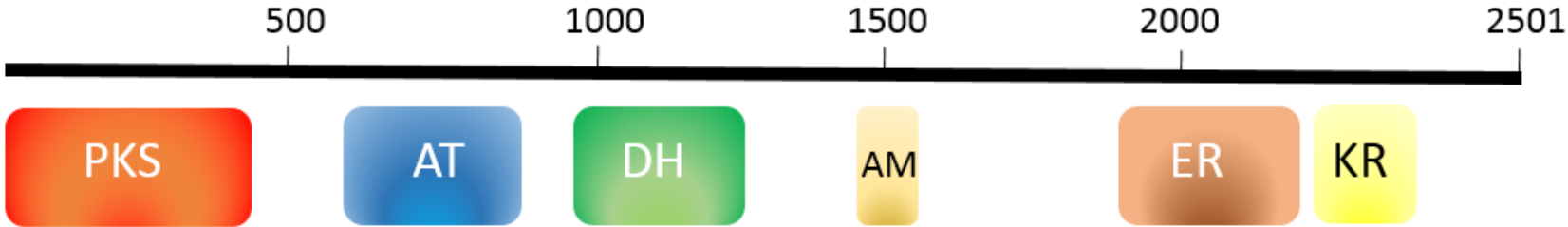

SOR2 = PKS10

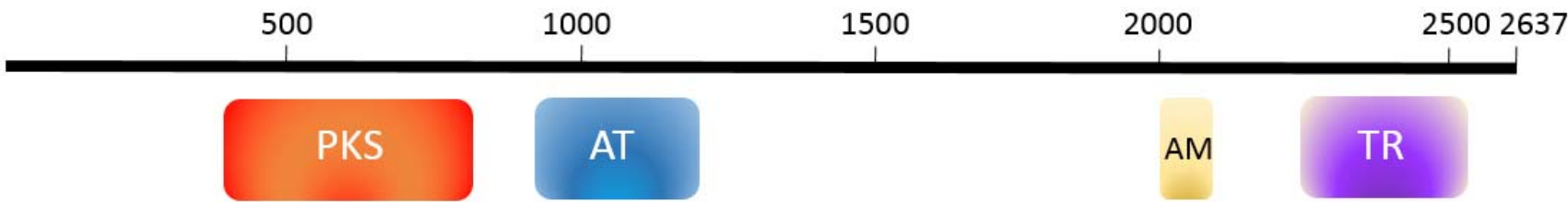

Supplement: Additional file 1: Figure S1. — Architecture of Trire2: 73618 and Trire2:73621. The bar specifies the size of the proteins (in amino acid residues). Abbreviations: PKS, polyketide synthase; AT, acyltransferase; DH, dehydrogenase; AM, adenosyl-methionine transferase; ER, enoyl reductase; KR, keto reductase; TR, thioester reductase. (PDF 165 kb) [file 12862_2016_834_MOESM1_ESM.pdf]

Additional file 2: Figure S2

A

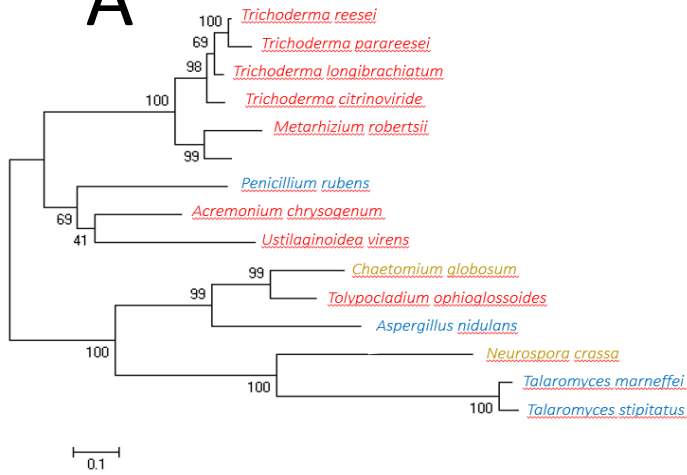

B

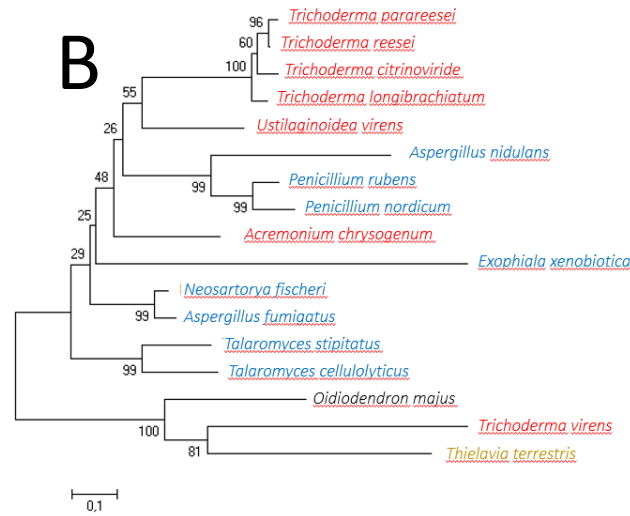

C

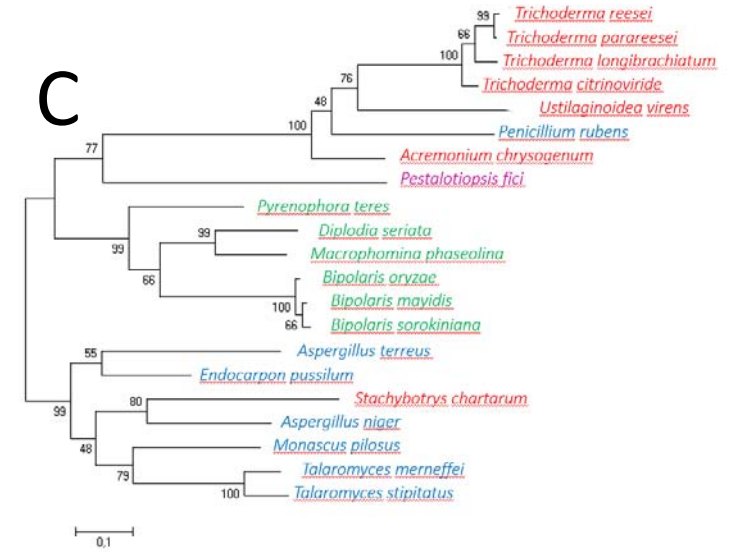

D

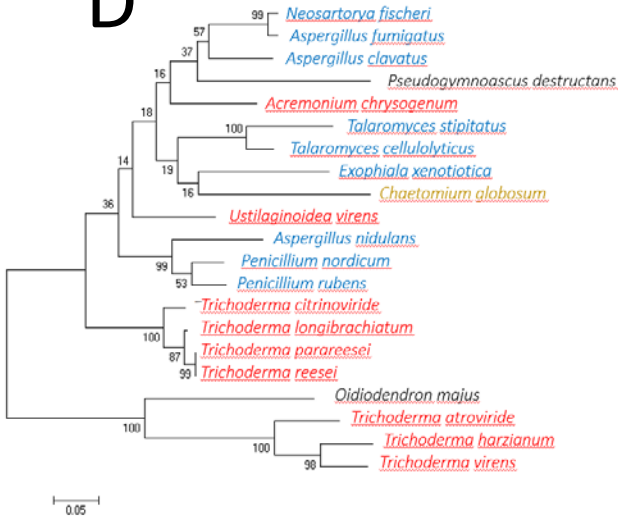

E

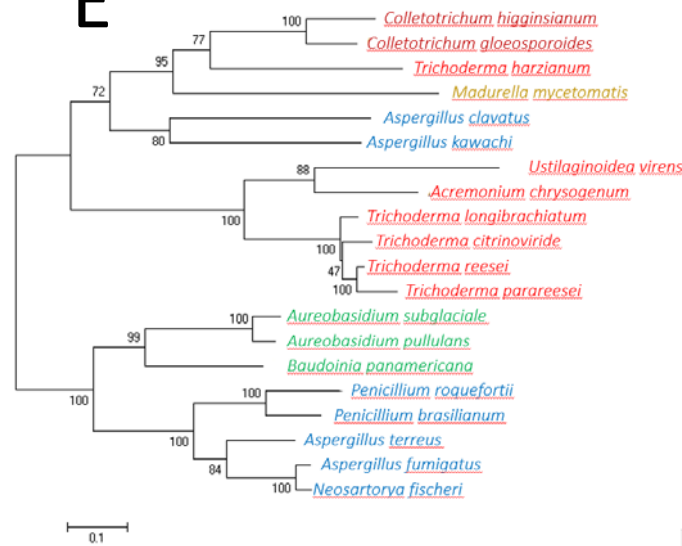

F

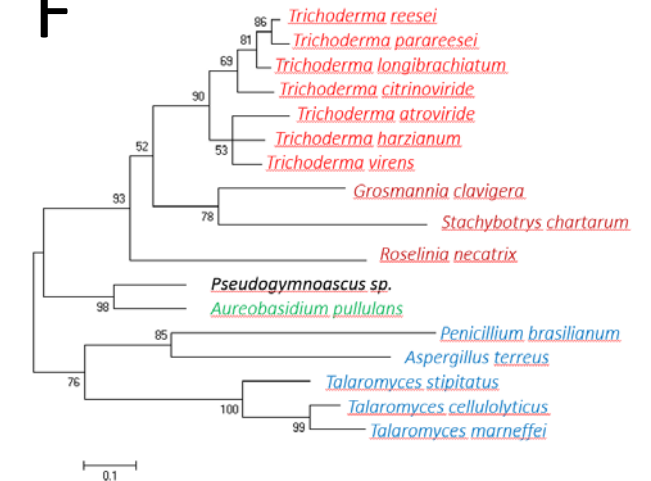

Supplement: Additional file 2: Figure S2. — Phylogenetic analysis of SOR3/SorC (A), SOR4/SorD (B), SOR5/SorE (C), SOR6/SorF (D), SOR7/SorG (E) and SOR8 (F) proteins by PhyML. Numbers at the nodes indicate the boostrap (1000 replicas) support. Numbers at the nodes indicate the bootstrap (1000 replicas) support. Colour codes are used as in Fig. 1. In addition, bright brown specifies members of the Sordariales. Accession numbers for all proteins shown are given in Additional file 9: Table S1. (PDF 368 kb) [file 12862_2016_834_MOESM2_ESM.pdf]

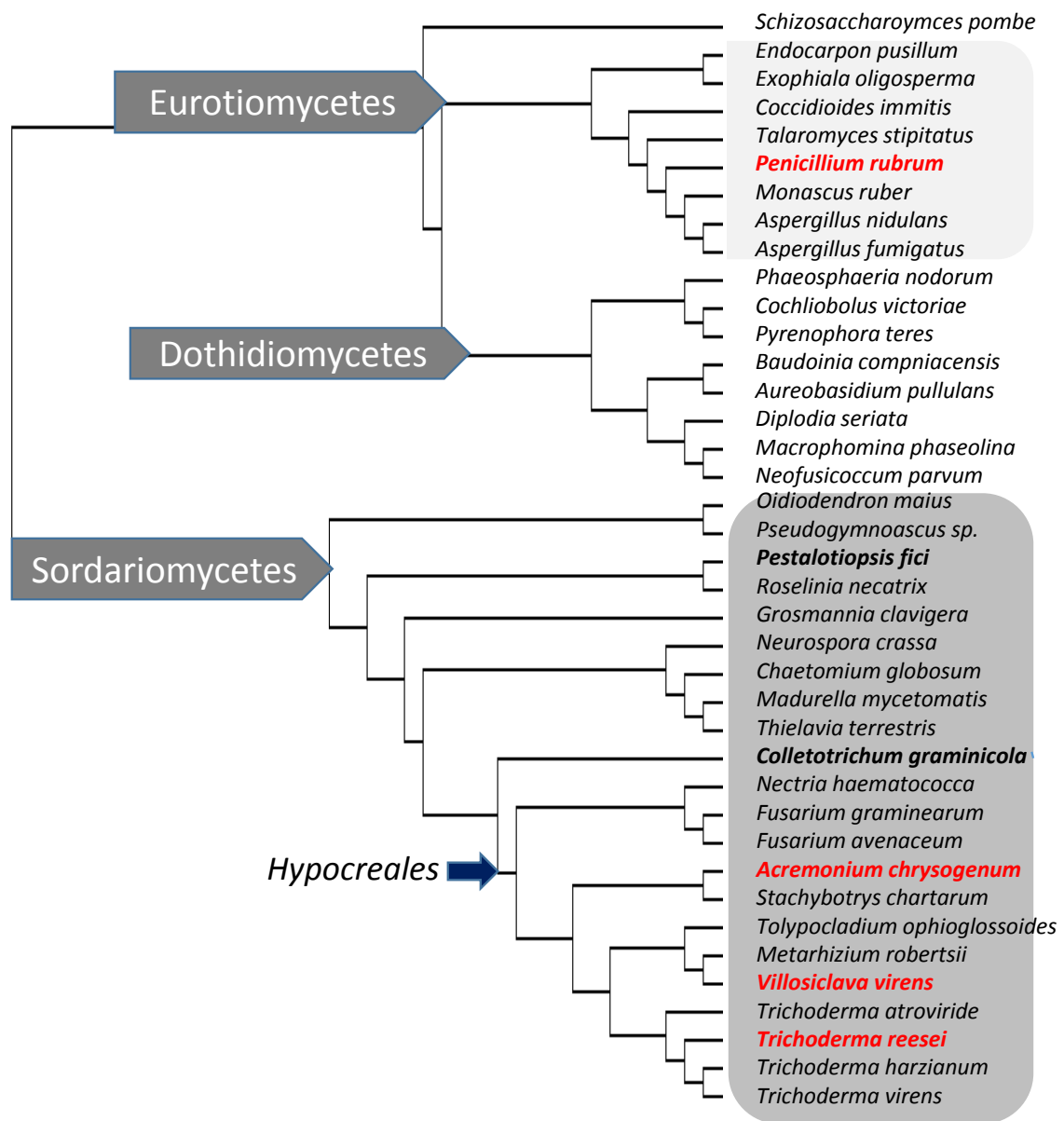

Additional file 3: Figure S3

Supplement: Additional file 3: Figure S3. — PhyML evolutionary tree of fungi, using protein sequences of the histone acetyltransferase subunit of RNA polymerase II, NAD-dependent glutamate dehydrogenase, translation initiation factor eIF-5, and Tsr1p, a protein required for processing of 20S pre-rRNA. For further details, see Methods. Species that contain a sorbicillinoid biosynthesis cluster are given in red. Donor species are printed in bold. Of Trichoderma, only T. reesei is shown for simplicity. (PDF 297 kb) [file 12862_2016_834_MOESM3_ESM.pdf]

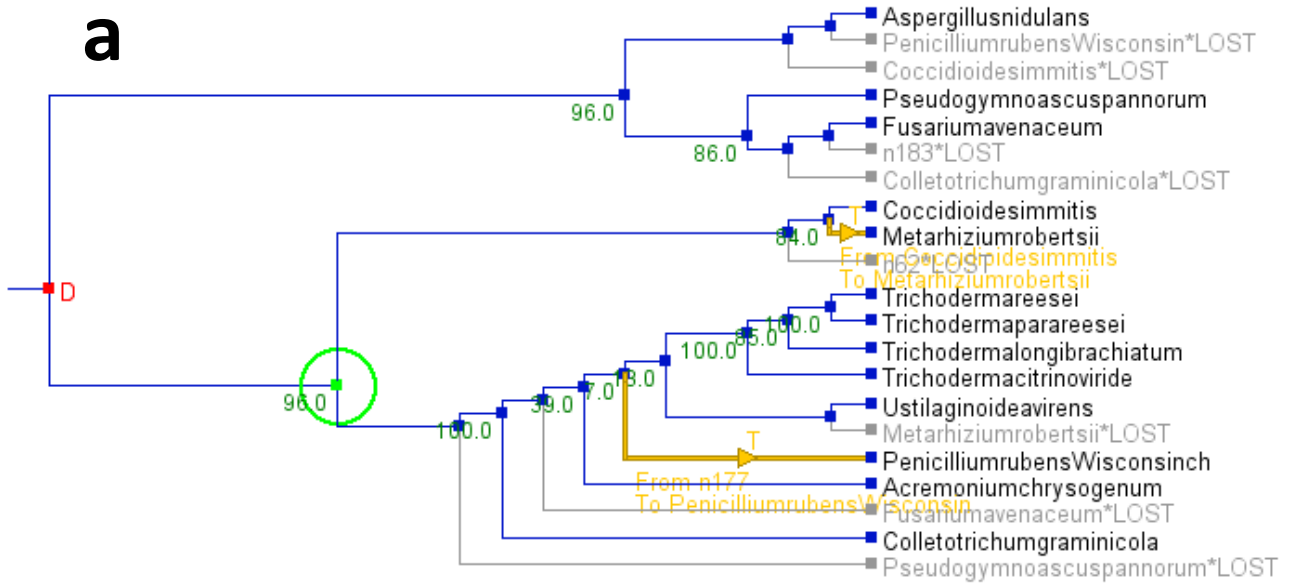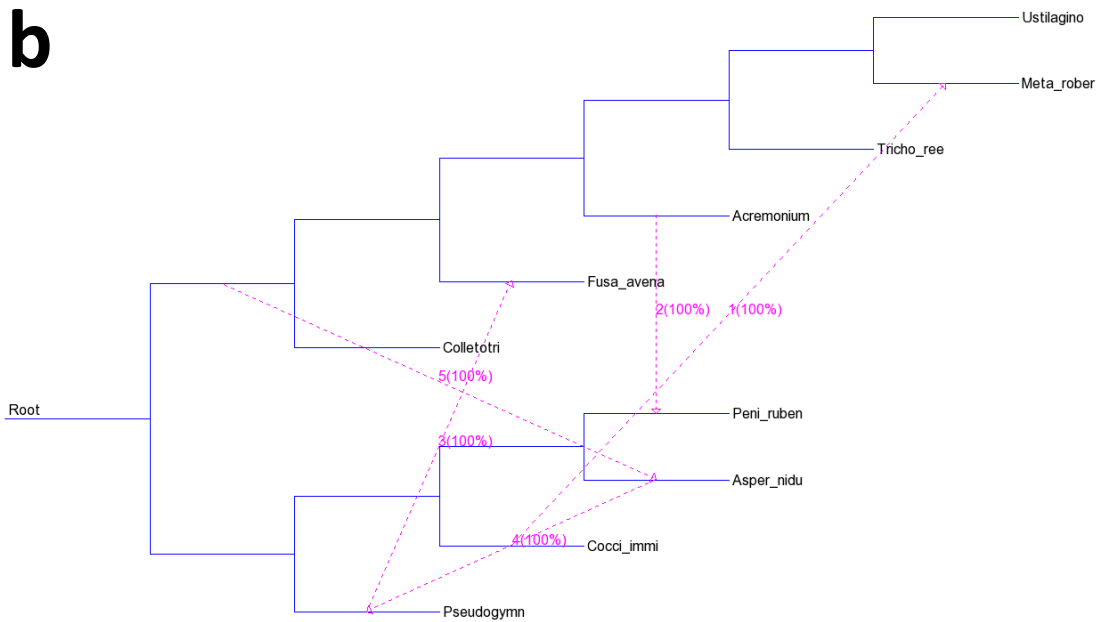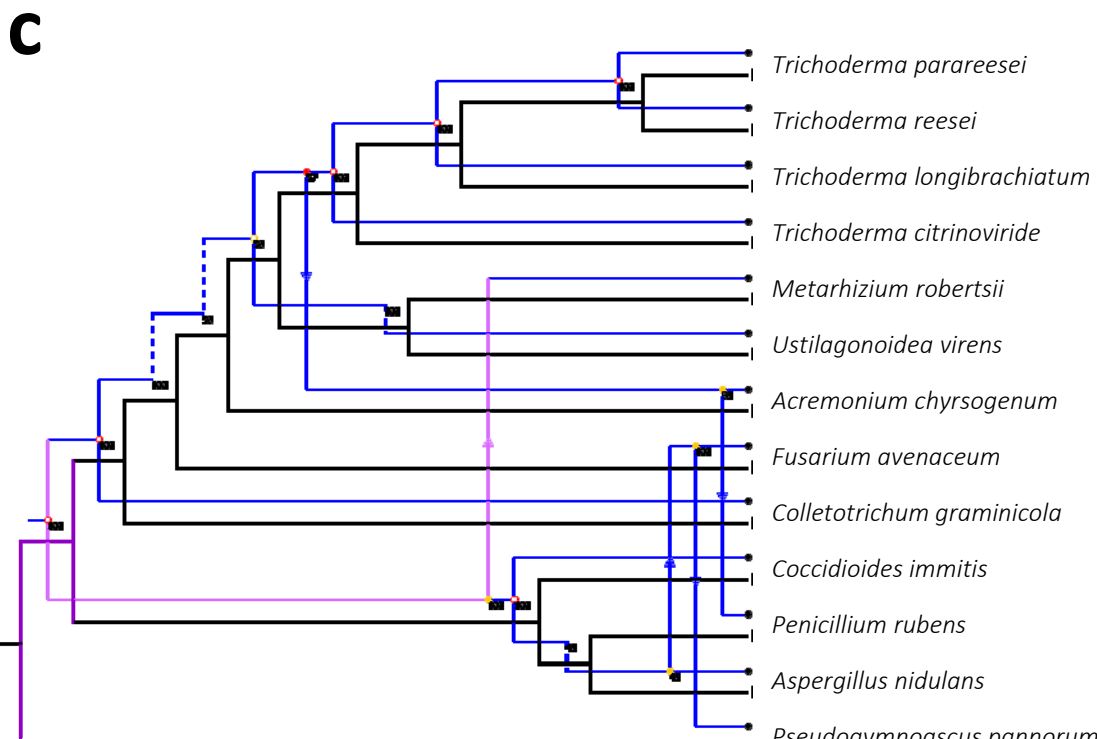

**a**

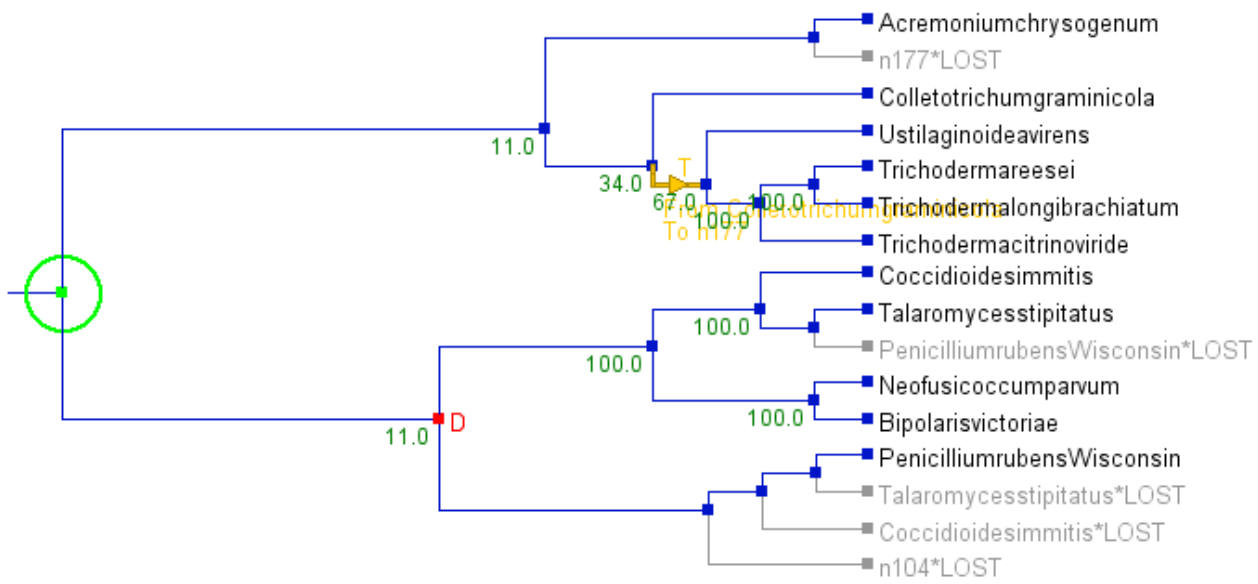

**b**

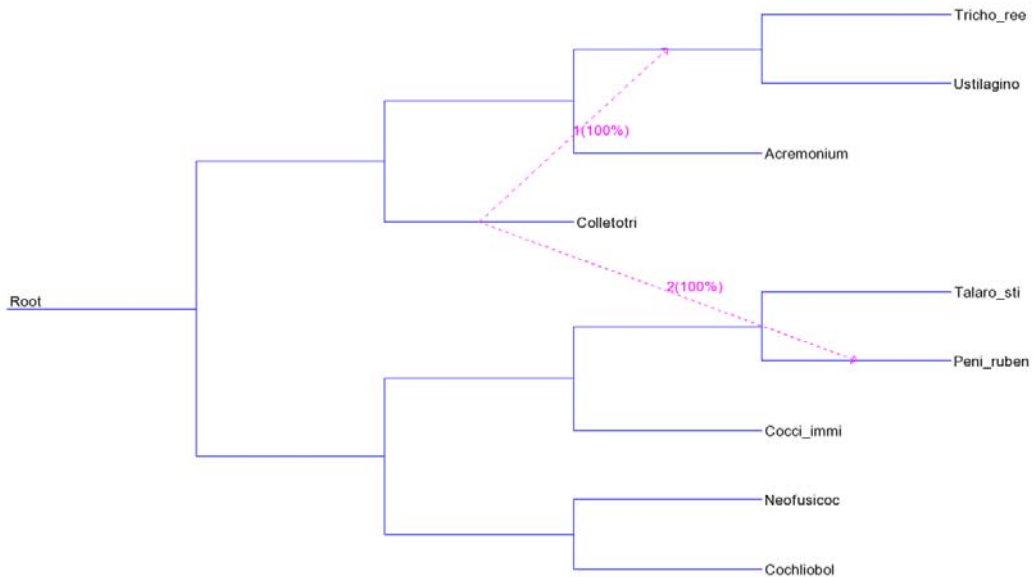

**c**

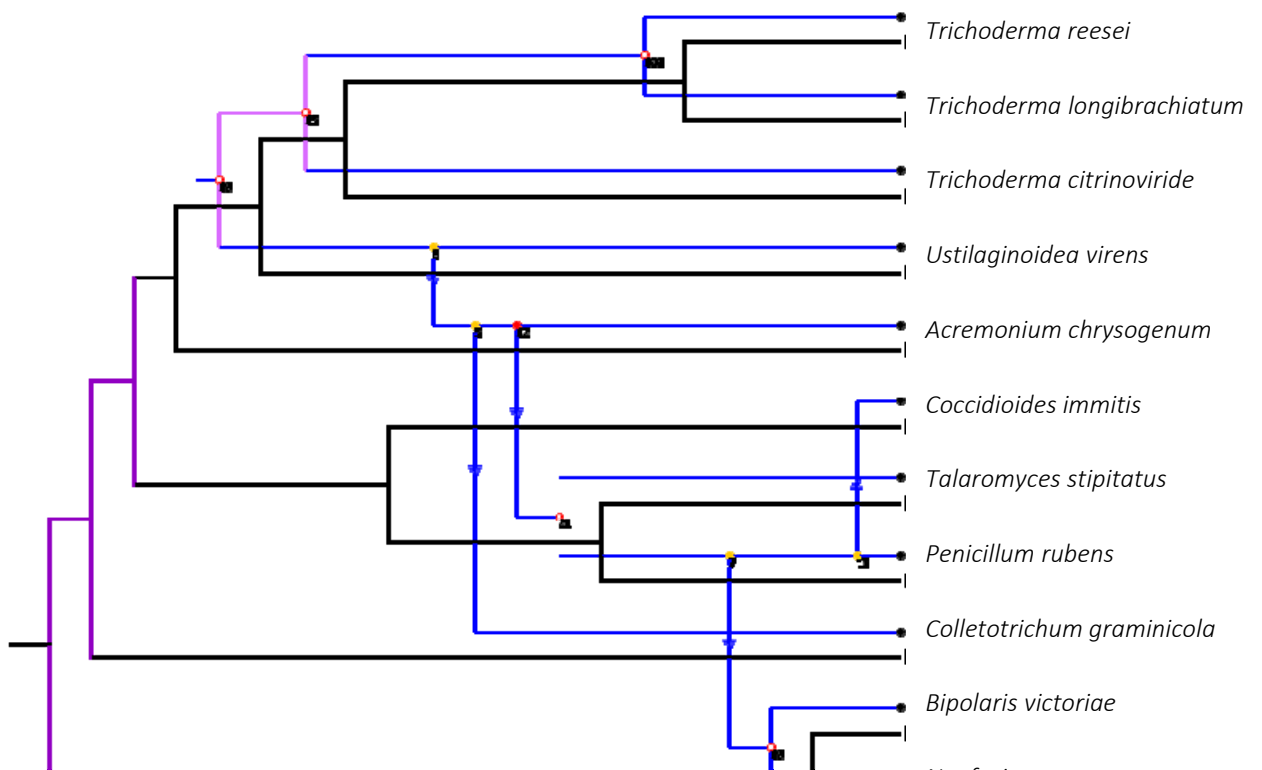

**a**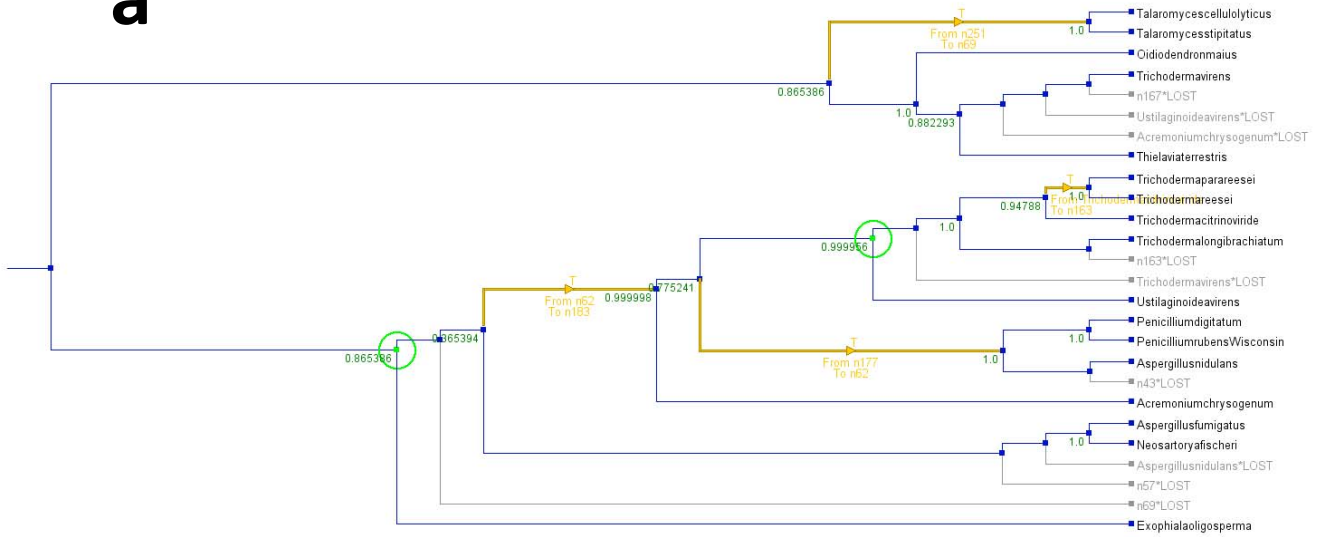**b**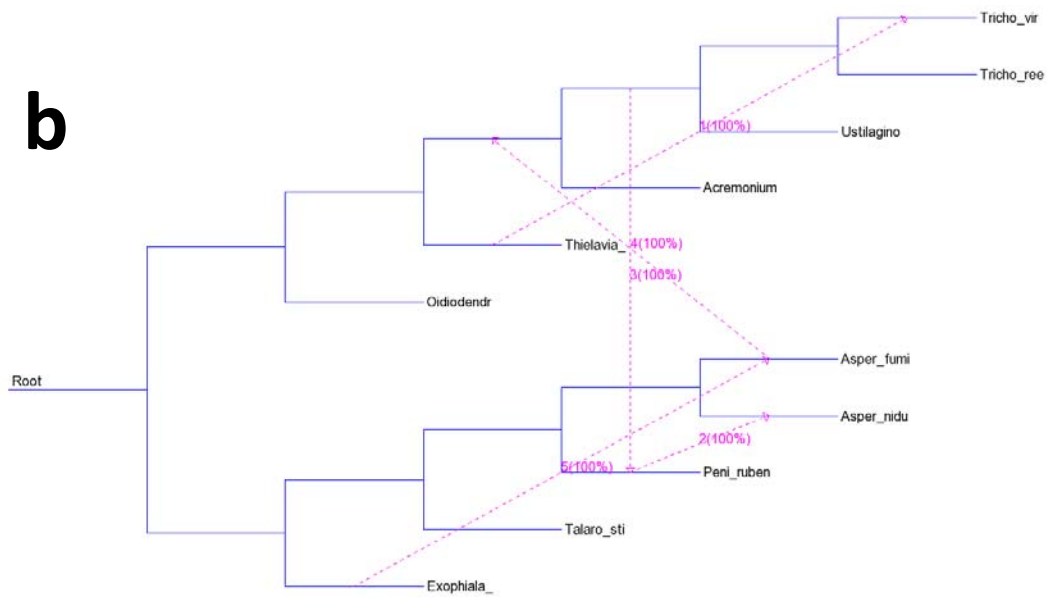**c**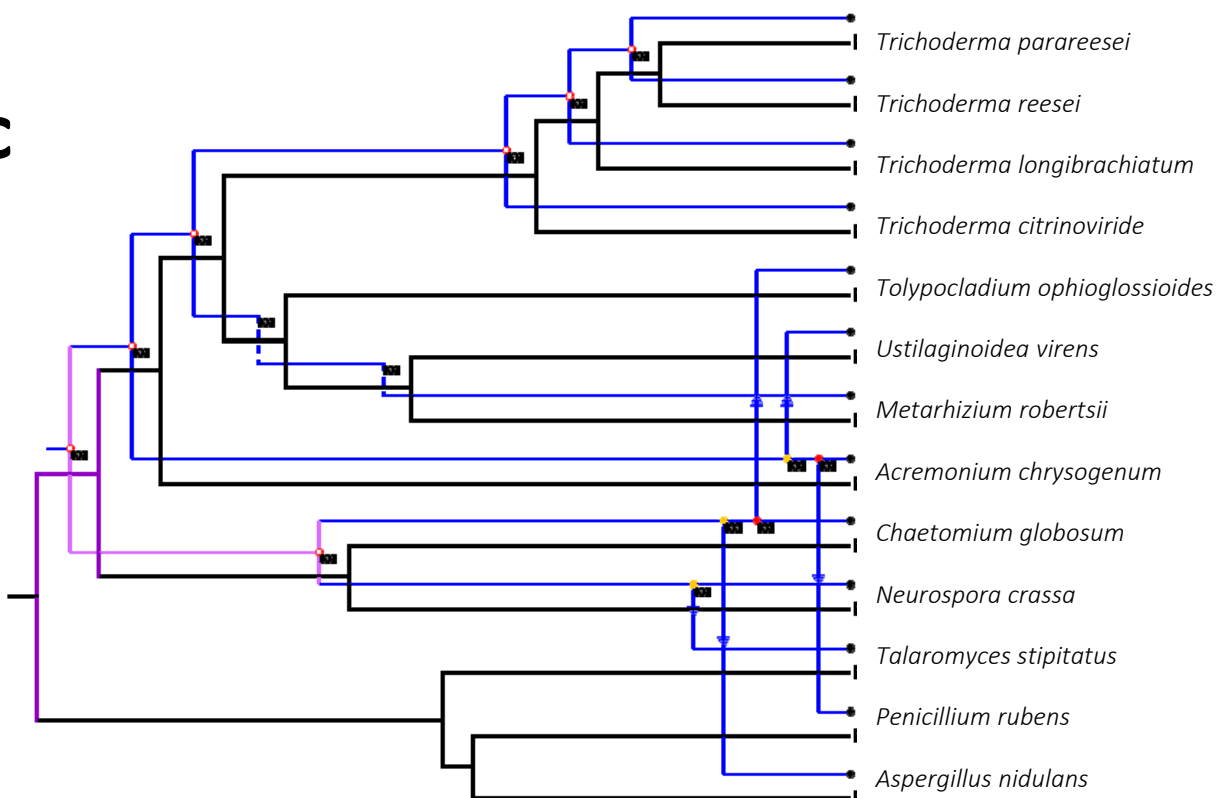

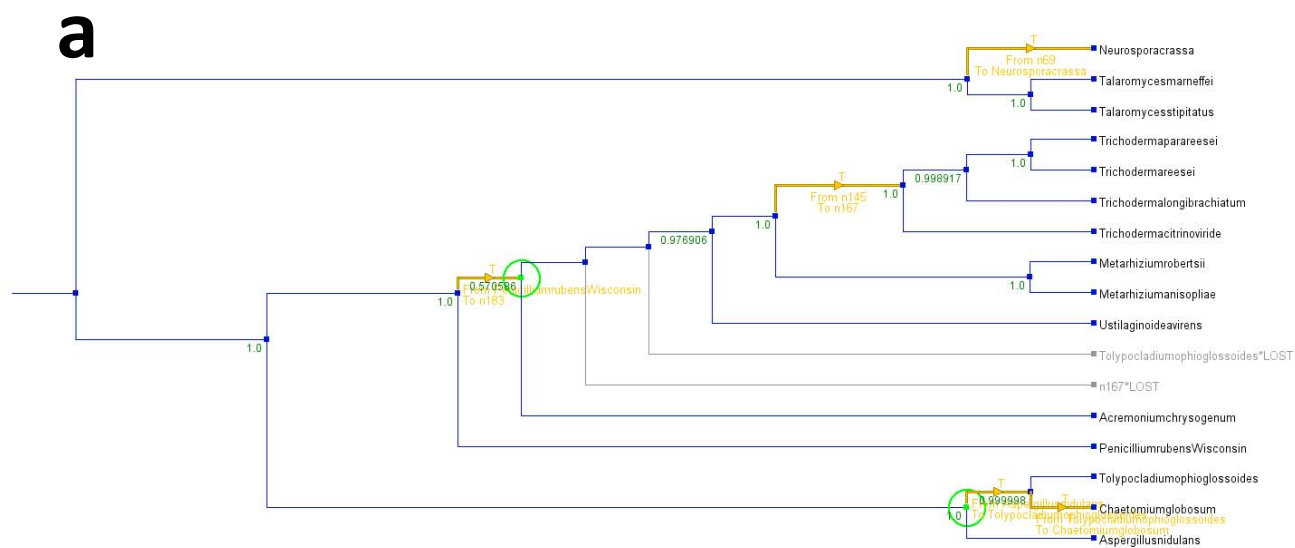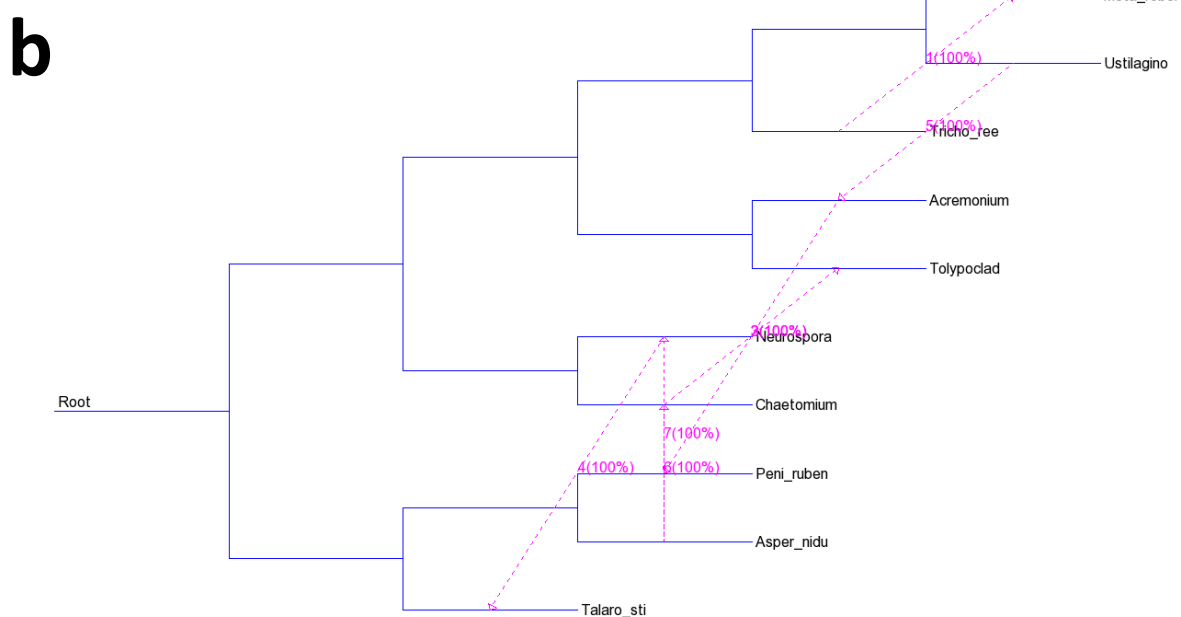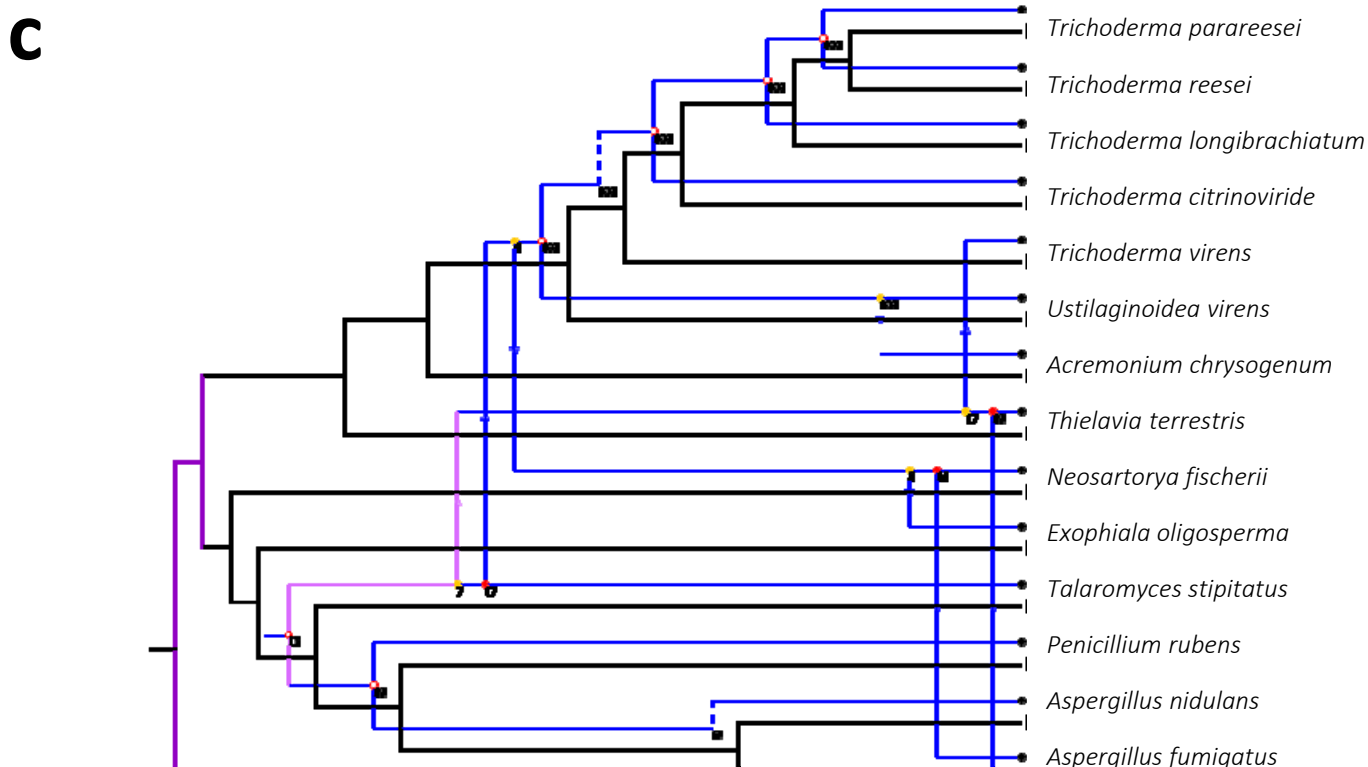

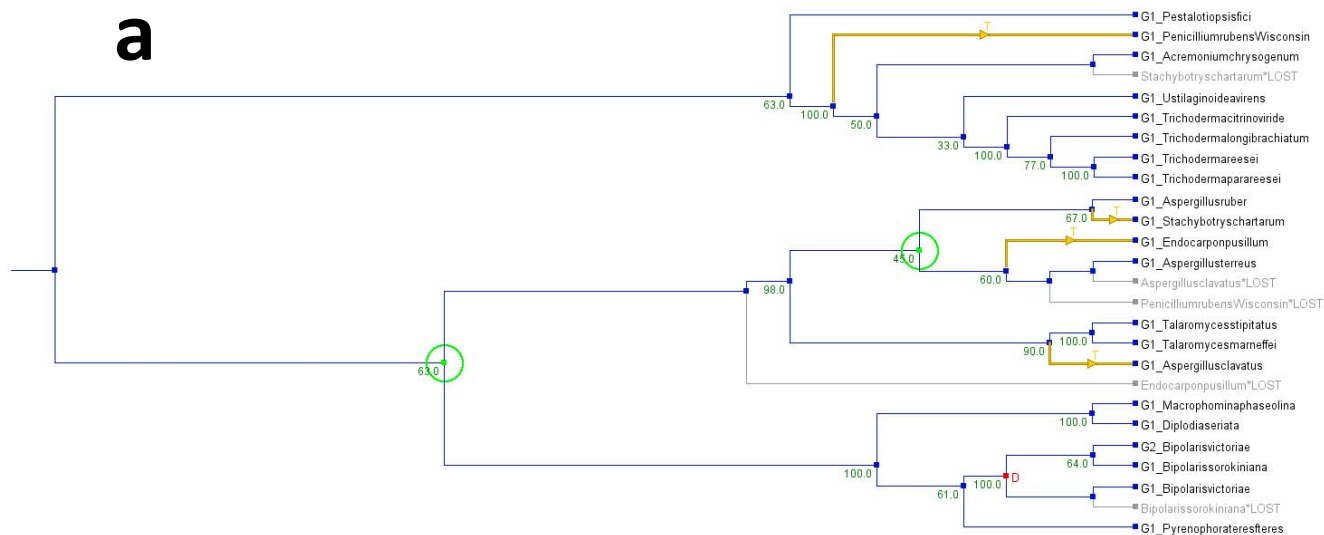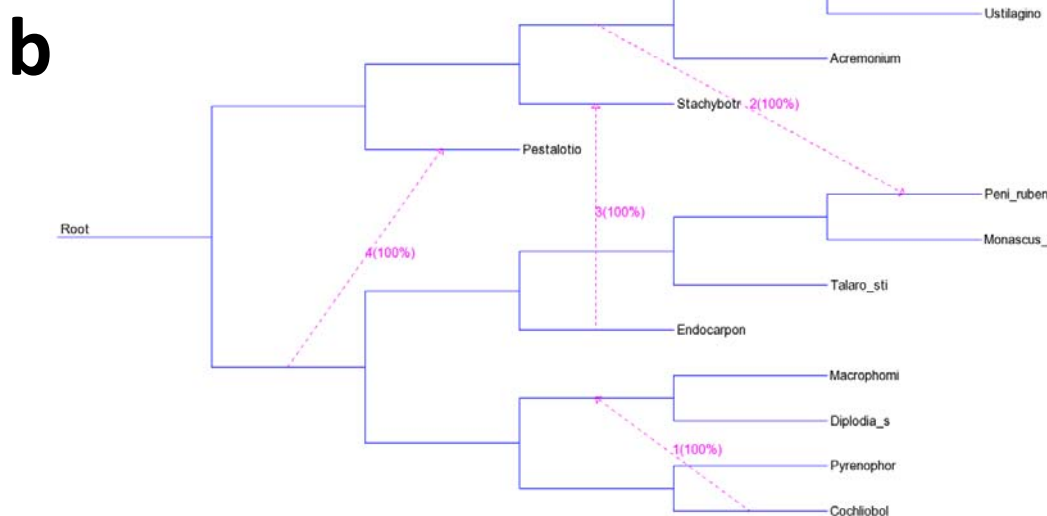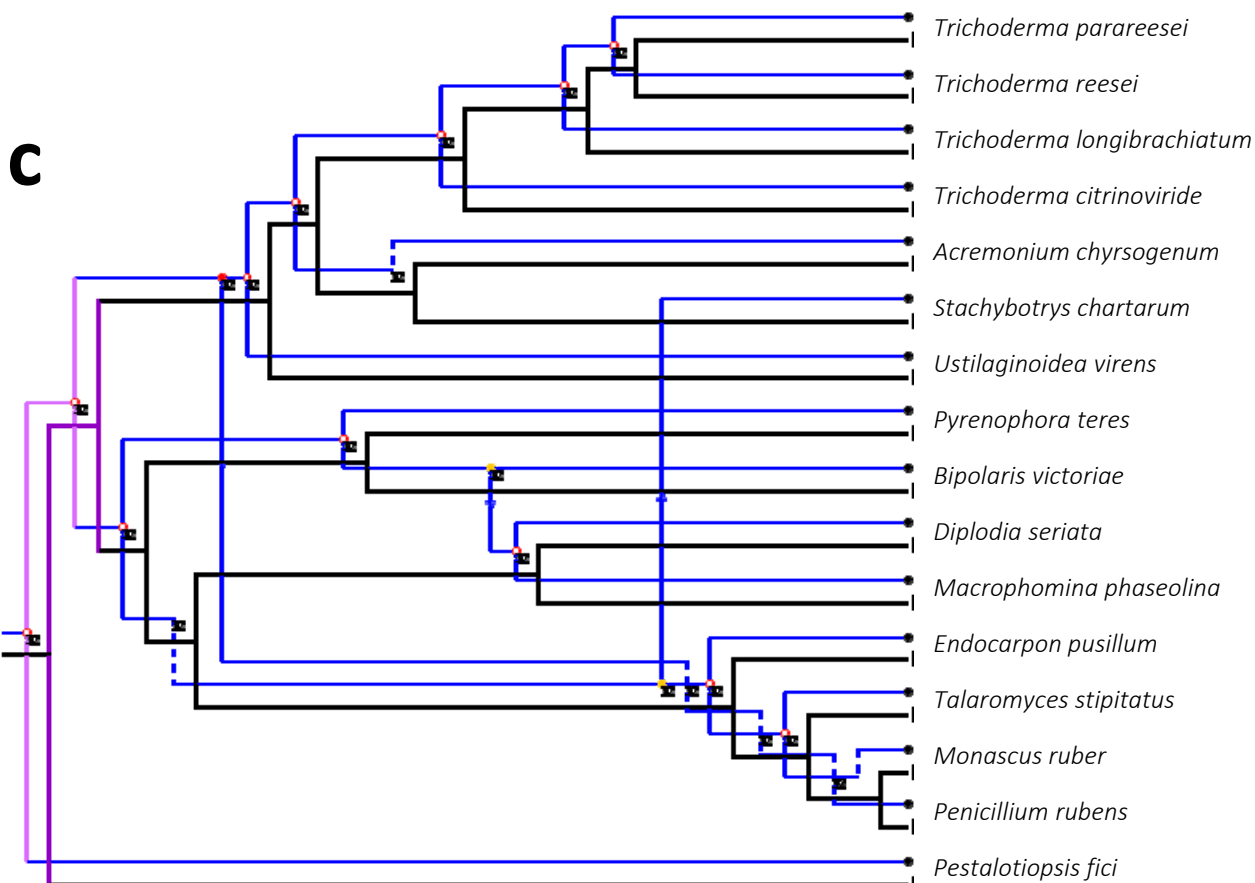

**a**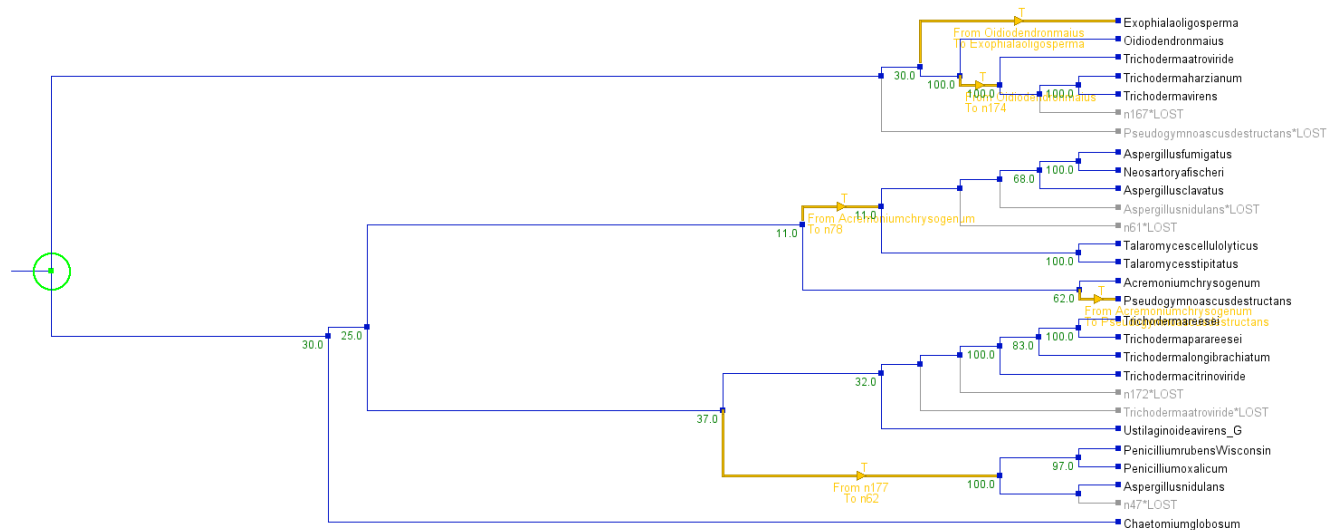**b**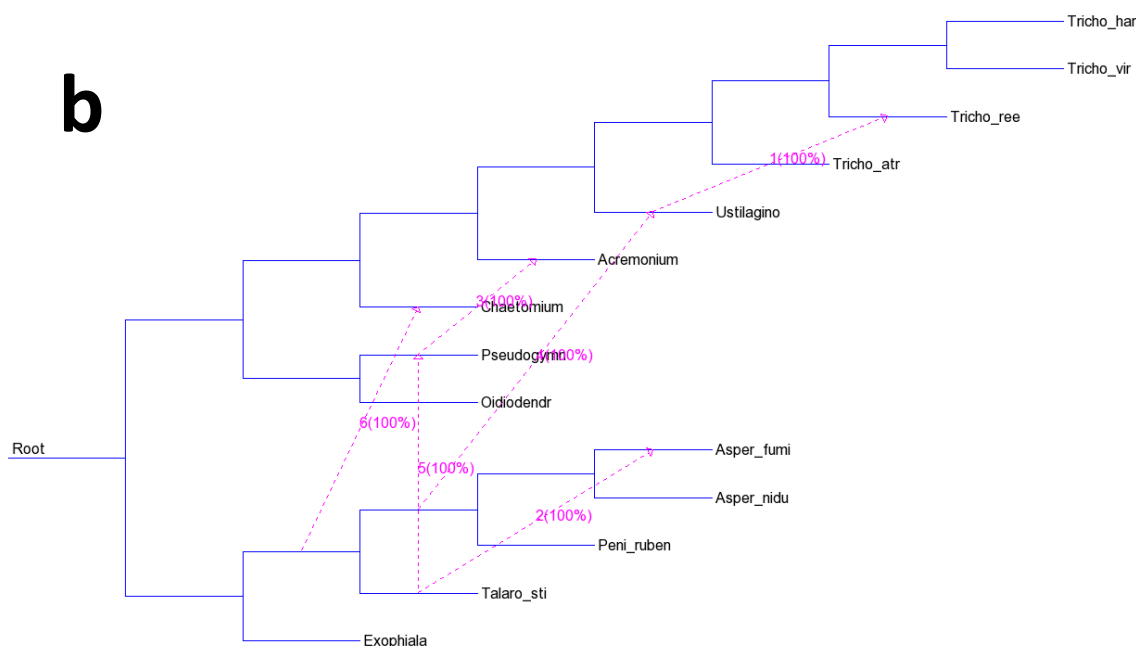**c**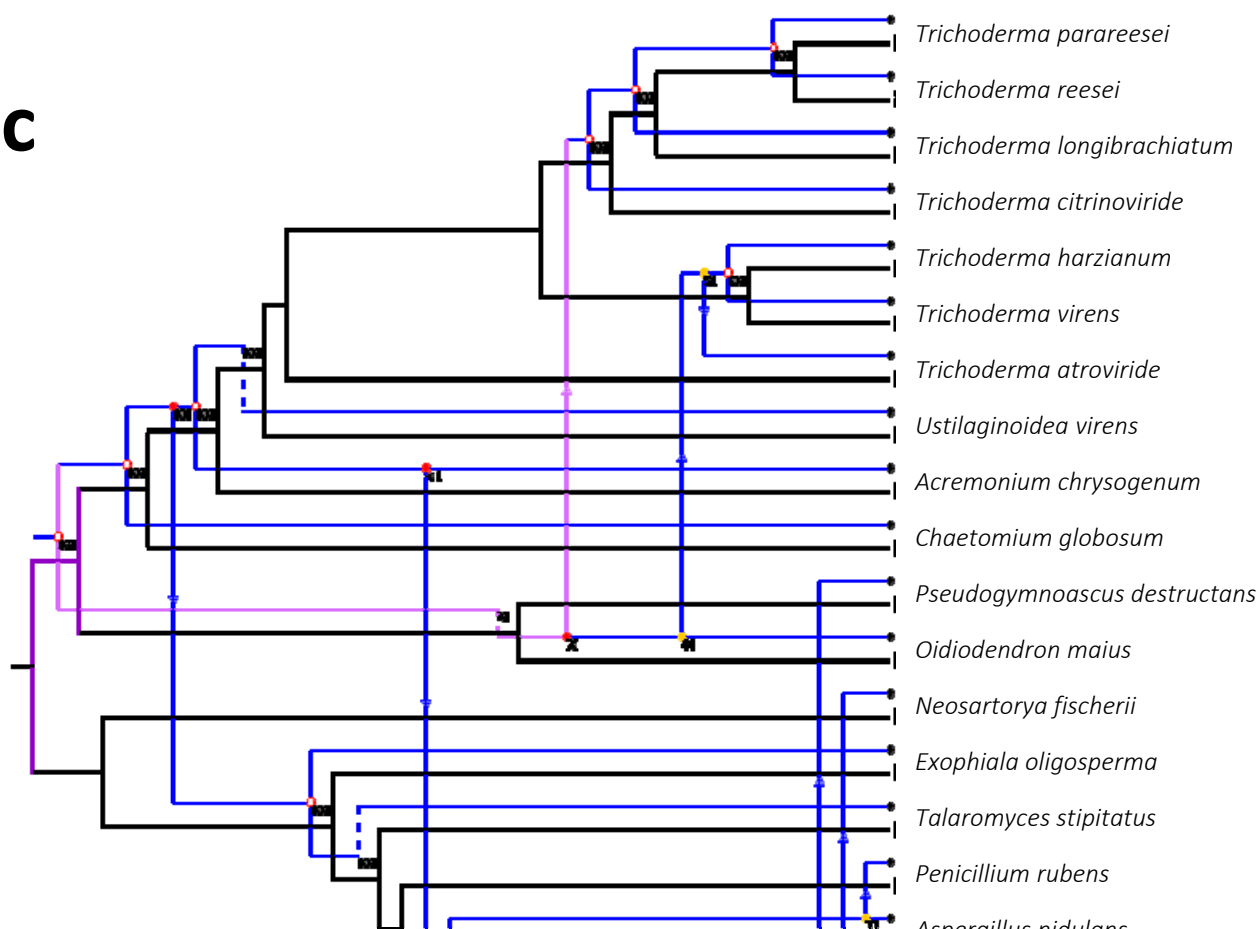

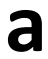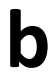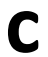

**a**

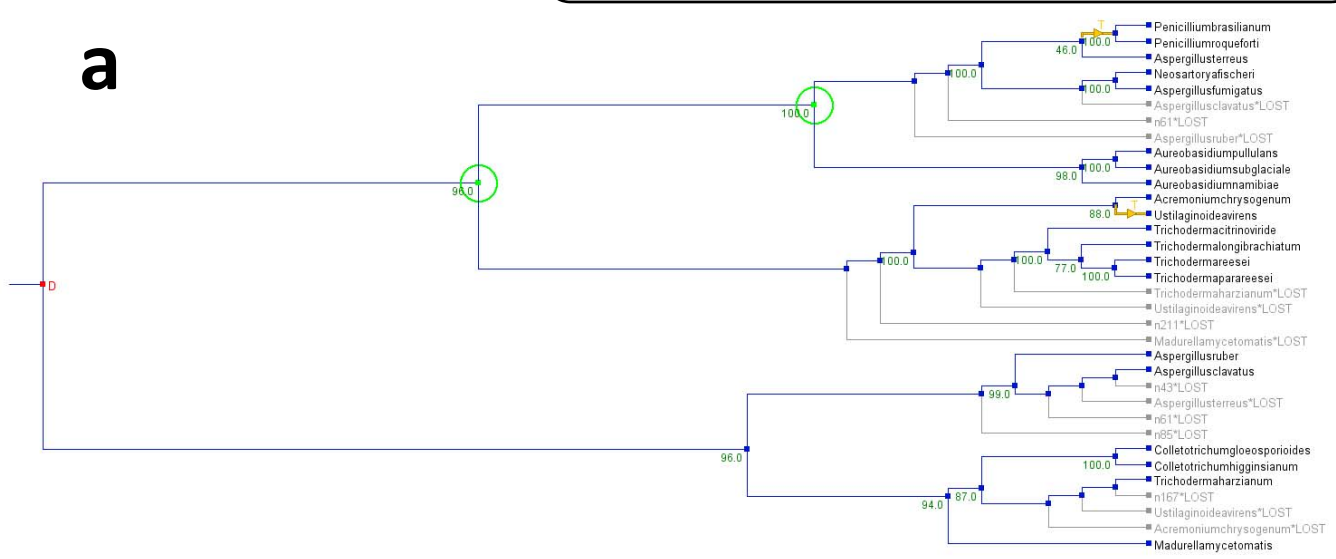

**b**

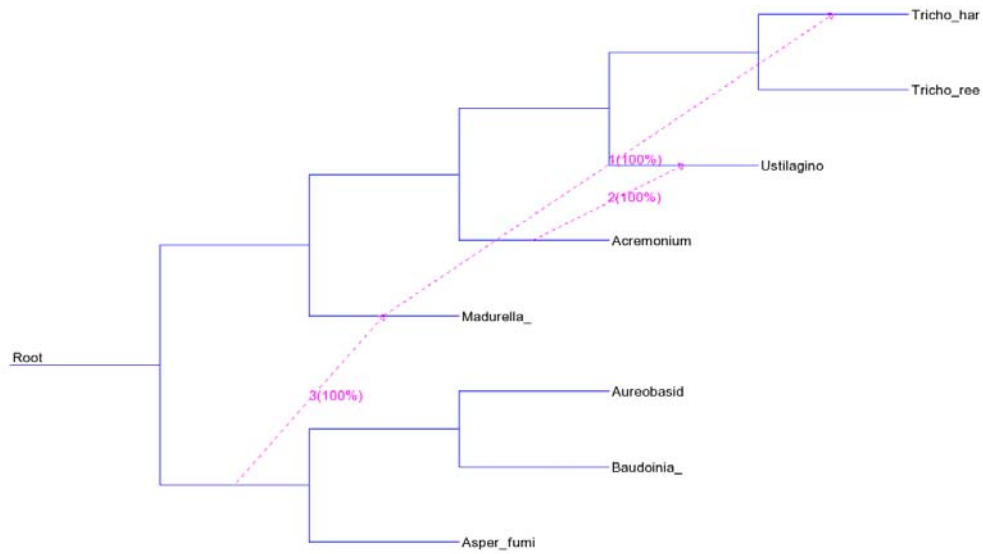

**c**

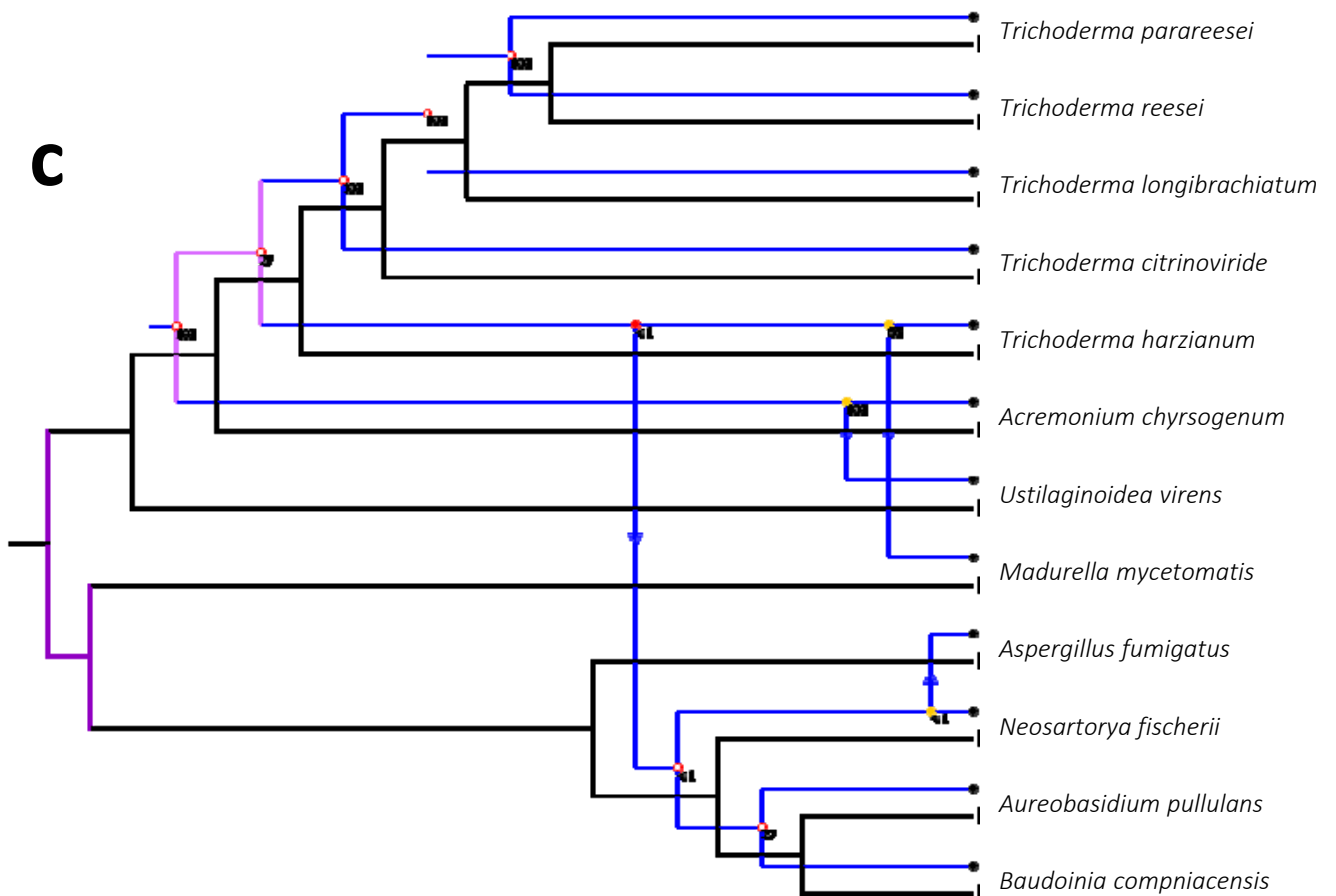

Supplement: Additional file 4: Figure S4. — Output trees of the analysis of SOR1-SOR8 by Notung (a), T-Rex (b) and Jane (c). In (a), yellow arrows indicate LGT, red D indicate duplication events; in (b), species names are abbreviated due to constraints of the program, but can easily be identified by comparing them to the species shown in (a) and (c); also note that of Trichoderma sect. Longibrachiatum, only T. reesei was used in these analyses; in (c), black lines identify the species tree, whereas blue lines indicate the protein tree. Lines with arrows show LGT, accompanied by support values. (PDF 673 kb) [file 12862_2016_834_MOESM4_ESM.pdf]

SOR1

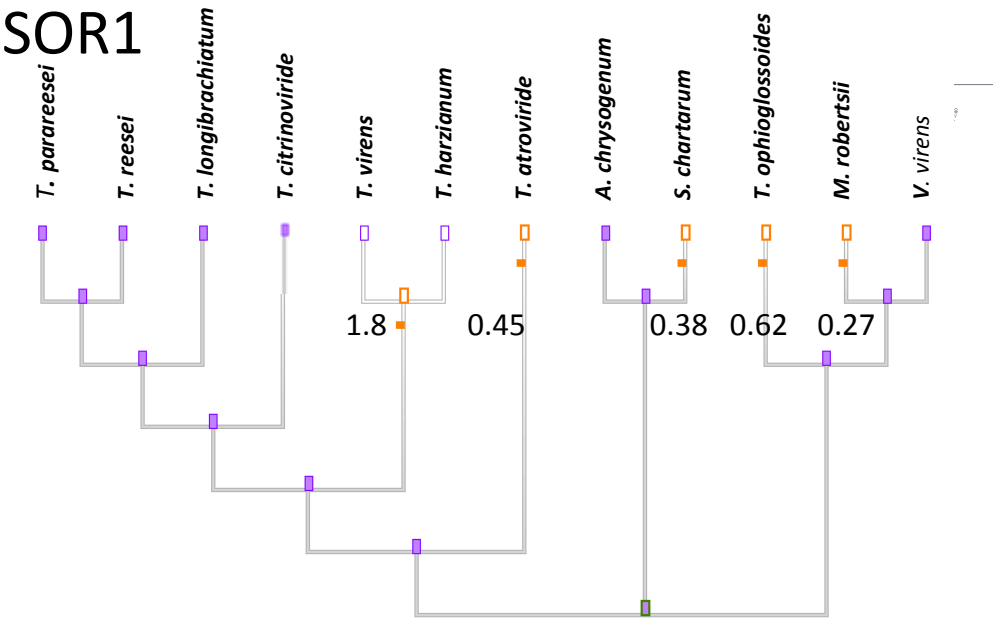

SOR2

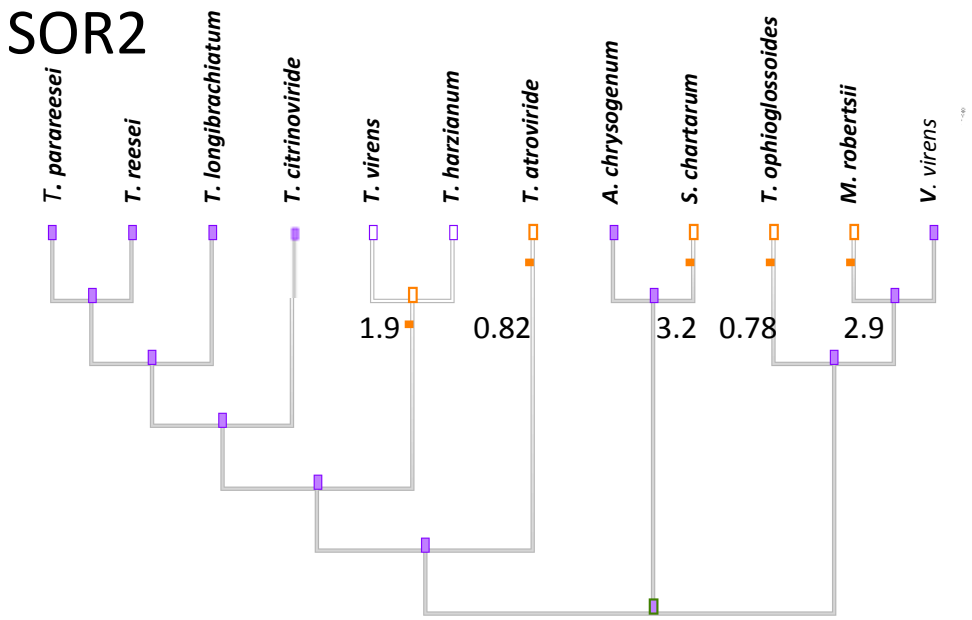

SOR3

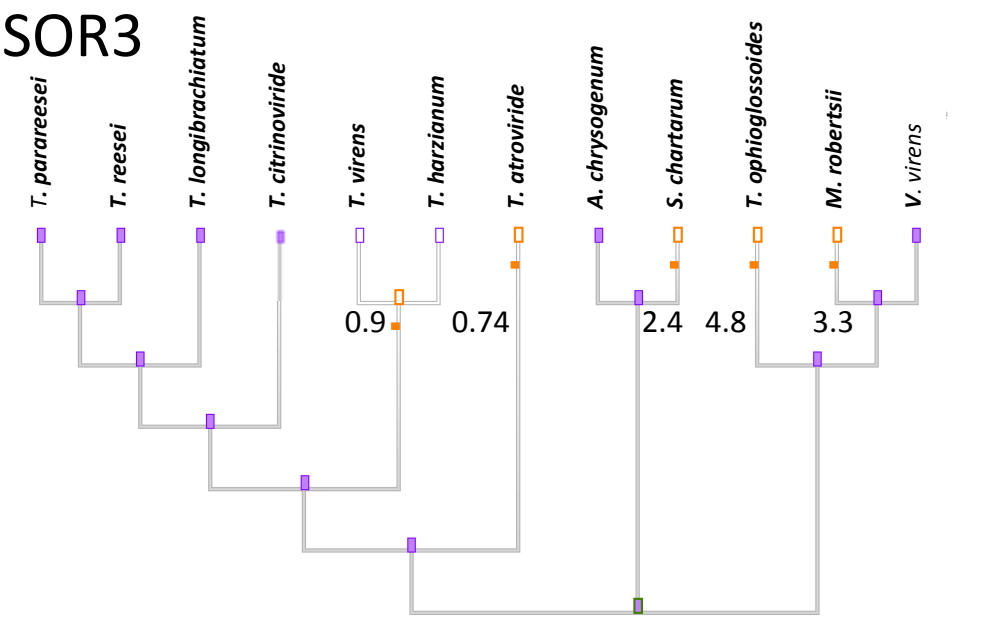

SOR4

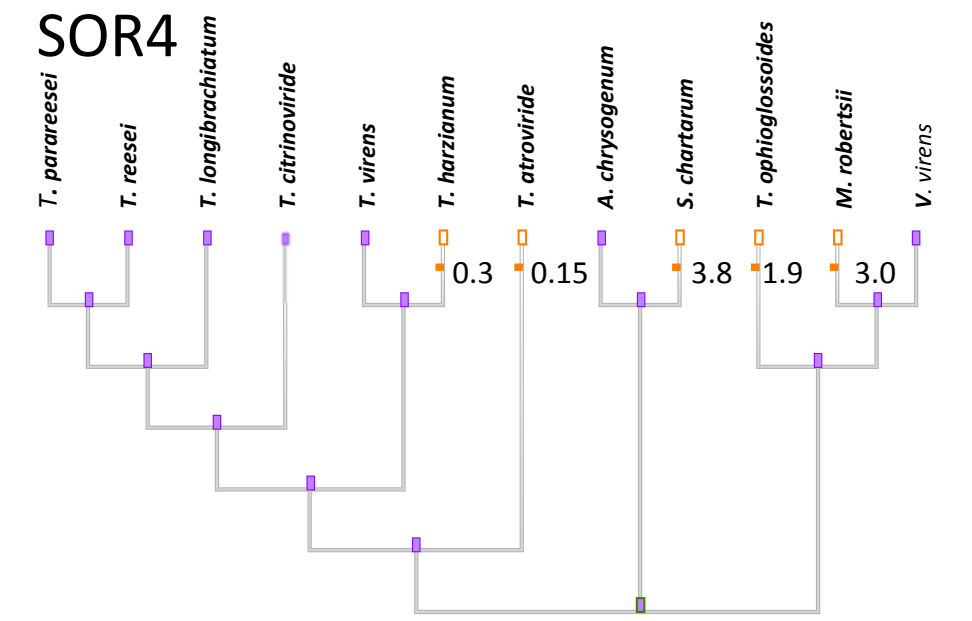

SOR5

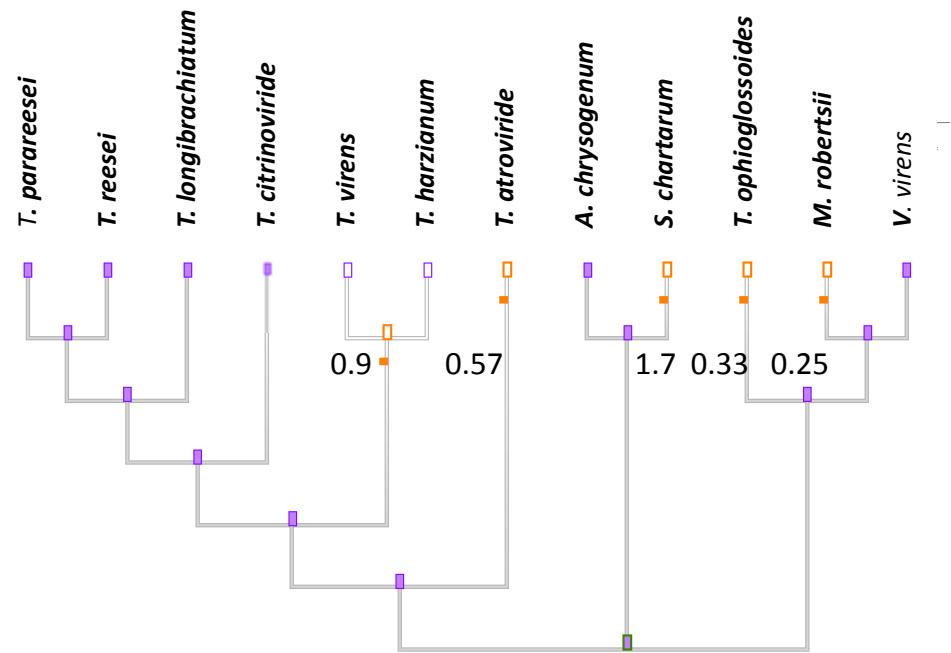

SOR6

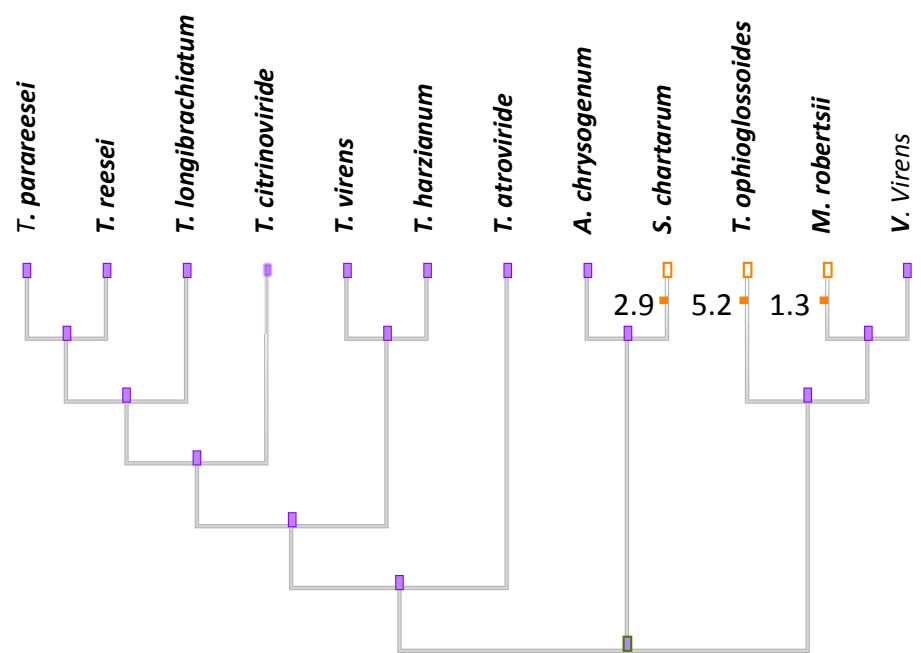

Supplement: Additional file 6: Figure S5. — Gain and loss of SOR1 – SOR6 in the Hypocreales. Open bars indicate gene loss, number at the nodes indicate the respective loss rates. (PDF 130 kb) [file 12862_2016_834_MOESM6_ESM.pdf]

Additional file 7: Figure S6

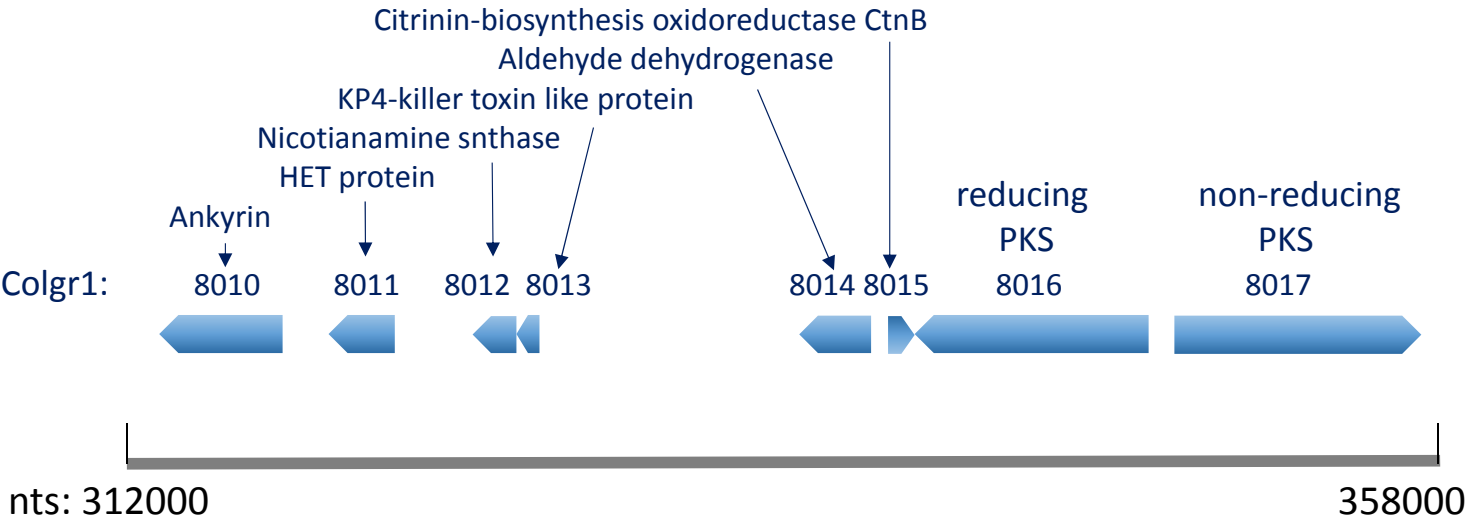

Supplement: Additional file 7: Figure S6. — Gene structure of the 3′ end of supercontig 46 of the C. graminicola genome sequence (http://genome.jgi.doe.gov/Colgr1/Colgr1.home.html). No further genes are located 3′ of Colgr1:8017. (PDF 219 kb) [file 12862_2016_834_MOESM7_ESM.pdf]
