# Supplementary material for: Several steps of lateral gene transfer followed by events of ‘birth-and-death’ evolution shaped a fungal sorbicillinoid biosynthetic gene cluster
Source: BMC Evol Biol. 2016 Dec 7;16:269. doi: 10.1186/s12862-016-0834-6 (PMC5182515; doi:10.1186/s12862-016-0834-6)
Supplement: Additional file 5: Table S2. — Statistics of T-Rex, Notung and Jane analyses. (DOCX 15 kb) [file 12862_2016_834_MOESM5_ESM.docx]

**Additional file 5: Table S2**: Statistics of T-Rex, Notung and Jane analyses.

| **Trire2:** | **T-Rex** | | | **Notung** | | | | | | |
| --- | --- | --- | --- | --- | --- | --- | --- | --- | --- | --- |
|  |  |  |  | *Duplications* | | | *Transfers* | | | |
|  | **Transfers** | **BD*** | **Bootstrap** | **score** | **dupl** | **loss** | **score** | **Transfers** | **dupl** | **loss** |
| 73618 | 5 | 17 | 100 | 100.5 | 2 | 90 | 15 | 4 | 0 | 3 |
| 73621 | 2 | 4.5 | 100 | 10 | 2 | 7 | 4 | 1 | 0 | 1 |
| 43701 | 6 | 22 | 100 | 61.5 | 5 | 54 | 27 | 8 | 0 | 3 |
| 73623 | 4 | 11 | 100 | 85.5 | 7 | 75 | 20 | 4 | 1 | 7 |
| 73631 | 3 | 2 | 100 | 49 | 4 | 3 | 15 | 4 | 0 | 3 |
| 102492 | 3 | 3.5 | 100 | 69.5 | 7 | 59 | 19.5 | 5 | 1 | 3 |
| 102497 | 5 | 14.5 | 100 | 66 | 6 | 57 | 24 | 5 | 0 | 9 |
| 102499 | 7 | 16.5 | 100 | 29 | 4 | 23 | 16 | 4 | 0 | 4 |
|  |  |  |  |  |  |  |  |  |  |  |
| *BD, bipartition dissimilarity | | |  |  |  |  |  |  |  |  |

**Jane**

|  | Species No. | transfers | Duplications | Duplication and transfer | loss | failure | costs | solutions |
| --- | --- | --- | --- | --- | --- | --- | --- | --- |
| 73618 | 13 | 7 | 0 | 5 | 4 | 0 | 14 | 20 |
| 73623 | 16 | 12 | 0 | 3 | 6 | 0 | 12 | 1000 |
| 73631 | 12 | 7 | 0 | 4 | 0 | 0 | 8 | 150 |
| 43701 | 18 | 9 | 0 | 8 | 3 | 0 | 19 | 116 |
| 102497 | 13 | 7 | 0 | 5 | 2 | 0 | 12 | 1000 |
| 102499 | 15 | 7 | 0 | 7 | 4 | 0 | 18 | 725 |
| 73621 | 11 | 5 | 0 | 5 | 0 | 0 | 10 | 330 |
| 102492 | 13 | 9 | 0 | 3 | 2 | 0 | 8 | 254 |
