# Supplementary material for: Several steps of lateral gene transfer followed by events of ‘birth-and-death’ evolution shaped a fungal sorbicillinoid biosynthetic gene cluster
Source: BMC Evol Biol. 2016 Dec 7;16:269. doi: 10.1186/s12862-016-0834-6 (PMC5182515; doi:10.1186/s12862-016-0834-6)
Supplement: Additional file 8: Table S3. — Trichodermol concentration in the culture fluid of T. reesei. (DOCX 12 kb) [file 12862_2016_834_MOESM8_ESM.docx]

**Additional file 8: Table S3.**

Trichodermol concentration in the culture fluid of *T. reesei**

1.78 [± 1.1] mg/L

n=6

* for quantification of trichodimerol as a marker metabolite of sorbicillinoids, *T. reesei* was grown on Mandels-Andreotti medium (Mandels and Andreotti 1978) with Avicel cellulose as carbon source. After 96 h cultivation, culture filtrates were harvested by filtration, and lyophilized. 2.5 g of the lyophilized samples were extracted with 14 mL of acetonitrile/water/acetic acid 79/20/1 for 90 min. An HPLC-ESI-MS/MS based multi-mycotoxin method using an Agilent 1290 HPLC coupled to an Applied Biosystems QTrap mass spectrometer was used for the analyses. Screening and Quantification were performed in the Selected Reaction Monitoring (SRM) mode, using the LC-MS/MS protocol of Vishwanat et al. (2009). 2 MRM (=multiple reaction monitoring) transitions were acquired per analyte, which yields 4 identification points for unambiguous identification

Mandels M, Andreotti RE (1978) Problems and challenges in the cellulose to cellulase fermentation. Process Biochem 13: 6–13

Vishwanath V, Sulyok M, Labuda R, Bicker W, Krska R. (2009) Simultaneous determination of 186 fungal and bacterial metabolites in indoor matrices by liquid chromatography/tandem mass spectrometry. Anal Bioanal Chem 395:1355-1372.
