# Supplementary material for: Several steps of lateral gene transfer followed by events of ‘birth-and-death’ evolution shaped a fungal sorbicillinoid biosynthetic gene cluster
Source: BMC Evol Biol. 2016 Dec 7;16:269. doi: 10.1186/s12862-016-0834-6 (PMC5182515; doi:10.1186/s12862-016-0834-6)
Supplement: Additional file 9: Table S1. — Accession numbers of proteins shown in Figs.1, 2 and 3. (DOCX 32 kb) [file 12862_2016_834_MOESM9_ESM.docx]

Additional file 9: Table S1. Proteins and their accession numbers used in this work.

| **Protein** | **Organism** | **Accession number*** | **Query cover** | **E-value** | **Similarity** |
| --- | --- | --- | --- | --- | --- |
|  |  |  | **%** |  | **%** |
| **SOR1/SorA** | *Trichoderma reesei* QM6a | ETS06290.1 | 100 | 0.0 | 100 |
|  | *Trichoderma parareesei* CBS125925 | g12738 | 100 | 0.0 | 100 |
|  | *Trichoderma longibrachiatum* ATCC 18648 | Trilo3:1326421 | 100 | 0.0 | 100 |
|  | *Trichoderma citrinoviride* | Trici4:1158066 | 100 | 0.0 | 100 |
|  | *Acremonium chrysogenum* ATCC 11550 | KFH44396.1 | 99 | 0.0 | 68 |
|  | *Penicillium rubens* Wisconsin 54-1255 | XP_002567554.1 | 99 | 0.0 | 65 |
|  | *Colletotrichum graminicola* M1.001 | XP_008098143.1 | 99 | 0.0 | 64 |
|  | *Fusarium avenaceum* | KIL88308.1 | 99 | 0.0 | 50 |
|  | *Tolypocladium ophioglossoides* | KND87310.1 | 99 | 0.0 | 51 |
|  | *Coccidioides immitis* RMSCC 2394 | KMP05705.1 | 98 | 0.0 | 51 |
|  | *Fusarium fujikuroi* IMI 58289 | CCT76008.1 | 99 | 0.0 | 49 |
|  | *Aspergillus oryzae* 3.042 | EIT81441.1 | 99 | 0.0 | 50 |
|  | *Aspergillus flavus* AF70 | KOC12412.1 | 99 | 0.0 | 50 |
|  | *Aspergillus nidulans* FGSC A4 | XP_664395.1 | 99 | 0.0 | 49 |
|  | *Metarhizium guizhouense* ARSEF 977 | KID84061.1 | 98 | 0.0 | 49 |
|  | *Pseudogymnoascus* sp. VKM F-4516 (FW-969) | KFY61065.1 | 99 | 0.0 | 48 |
|  |  |  |  |  |  |
| **SOR2/SorB** | *Trichoderma reesei* QM6a | XP_006961156.1 | 100 | 0.0 | 100 |
|  | *Trichoderma parareesei* CBS125925 | g12735 | 100 | 0.0 | 100 |
|  | *Trichoderma longibrachiatum* ATCC 18648 | Trilo3:1419916 | 100 | 0.0 | 100 |
|  | *Trichoderma citrinoviride* | Trici4:1151865 | 100 | 0.0 | 100 |
|  | *Acremonium chrysogenum* ATCC 11550 | KFH44362.1 | 99 | 0.0 | 65 |
|  | *Penicillium rubens* Wisconsin 54-1255 | XP_002567553.1 | 100 | 0.0 | 65 |
|  | *Ustilaginoidea virens* | KDB13139.1 | 99 | 0.0 | 64 |
|  | *Colletotrichum graminicola* M1.001 | XP_008098144.1 | 99 | 0.0 | 59 |
|  | *Neofusicoccum parvum* UCRNP2 | XP_007579490.1 | 98 | 0.0 | 48 |
|  | *Talaromyces cellulolyticus* | GAM33949.1 | 99 | 0.0 | 46 |
|  | *Talaromyces marneffei* ATCC 18224 | XP_002149737.1 | 99 | 0.0 | 46 |
|  | *Talaromyces stipitatus* ATCC 10500 | XP_002340070.1 | 99 | 0.0 | 46 |
|  | *Coccidioides immitis* RS | XP_001243185.2 | 99 | 0.0 | 44 |
|  | *Bipolaris victoriae* FI3 | XP_014551908.1 | 99 | 0.0 | 45 |
|  |  |  |  |  |  |
| **SOR3/SorC** | *Trichoderma reesei* QM6a | ETS06296.1 | 100 | 0.0 | 100 |
|  | *Trichoderma parareesei* CBS125925 | g12728 | 100 | 0.0 | 100 |
|  | *Trichoderma longibrachiatum* ATCC 18648 | Trilo3:1371623 | 100 | 0.0 | 100 |
|  | *Trichoderma citrinoviride* | Trici4:1128374 | 100 | 0.0 | 100 |
|  | *Metarhizium robertsii* | EXU99154.1 | 99 | 0.0 | 68 |
|  | *Acremonium chrysogenum* ATCC 11550 | KFH44401.1 | 96 | 0.0 | 49 |
|  | *Ustilaginoidea virens* | KDB13138.1 | 96 | 8,00E-169 | 46 |
|  | *Penicillium rubens* Wisconsin 54-1255 | XP_002567551.1 | 95 | 1,00E-149 | 42 |
|  | *Chaetomium globosum* CBS 148.51 | XP_001225298.1 | 95 | 9,00E-111 | 36 |
|  | *Tolypocladium ophioglossoides* CBS 100239 | KND87343.1 | 94 | 5,00E-109 | 39 |
|  | *Talaromyces stipitatus* ATCC 10500 | XP_002487359.1 | 95 | 1,00E-84 | 31 |
|  | *Neurospora crassa* OR74A | XP_959123.2 | 96 | 1,00E-81 | 35 |
|  | *Talaromyces marneffei* ATCC 18224 | XP_002149768.1 | 95 | 3,00E-75 | 31 |
|  | fungal specific transcription factor domain protein [*Aspergillus clavatus* NRRL 1] | XP_001276438.1 | 94 | 8,00E-75 | 31 |
|  |  |  |  |  |  |
| **SOR4/SorD** | *Trichoderma reesei* QM6a | ETS06294.1 | 100 | 0.0 | 100 |
|  | *Trichoderma parareesei* CBS125925 | g12731 | 100 | 0.0 | 100 |
|  | *Trichoderma longibrachiatum* ATCC 18648 | Trilo3:1398856 | 100 | 0.0 | 100 |
|  | *Trichoderma citrinoviride* | Trici4:1124242 | 100 | 0.0 | 100 |
|  | *Neosartorya fischeri* NRRL 181 | XP_001258840.1 | 98 | 0.0 | 60 |
|  | *Ustilaginoidea virens* | KDB13141.1 | 98 | 0.0 | 58 |
|  | *Penicillium rubens* Wisconsin 54-1255 | XP_002567555.1 | 98 | 0.0 | 57 |
|  | *Penicillium nordicum* | KOS45577.1 | 98 | 0.0 | 56 |
|  | *Acremonium chrysogenum* ATCC 11550 | KFH44394.1 | 98 | 1,00E-180 | 54 |
|  | *Talaromyces stipitatus* ATCC 10500 | XP_002486698.1 | 97 | 1,00E-176 | 53 |
|  | *Talaromyces cellulolyticus* | GAM41975.1 | 97 | 3,00E-173 | 53 |
|  | *Aspergillus nidulans* FGSC A4 | XP_660873.1 | 98 | 2,00E-156 | 48 |
|  | *Oidiodendron maius* Zn | KIN00607.1 | 99 | 1,00E-127 | 39 |
|  | *Exophiala xenobiotica* | XP_013322436.1 | 61 | 5,00E-98 | 53 |
|  | *Trichoderma virens* Gv29-8 | XP_013956237.1 | 97 | 6,00E-98 | 38 |
|  |  |  |  |  |  |
| **SOR5/SorE** | *Trichoderma reesei* QM6a | ETS06292.1 | 100 | 0.0 | 100 |
|  | *Trichoderma parareesei* CBS125925 | g12733 | 100 | 0.0 | 100 |
|  | *Trichoderma longibrachiatum* ATCC 18648 | Trilo3:1350419 | 100 | 0.0 | 100 |
|  | *Trichoderma citrinoviride* | Trici4:59919 | 100 | 0.0 | 100 |
|  | *Acremonium chrysogenum* ATCC 11550 | KFH44392.1 | 97 | 0.0 | 62 |
|  | *Penicillium rubens* Wisconsin 54-1255 | XP_002567552.1 | 96 | 0.0 | 58 |
|  | *Ustilaginoidea virens* | KDB13146.1 | 97 | 4,00E-179 | 59 |
|  | *Macrophomina phaseolina* MS6 | EKG11421.1 | 95 | 5,00E-121 | 44 |
|  | *Diplodia seriata* | KKY14251.1 | 95 | 2,00E-118 | 43 |
|  | *Talaromyces marneffei* ATCC 18224 | XP_002149772.1 | 94 | 1,00E-108 | 41 |
|  | *Stachybotrys chartarum* IBT 40288 | KFA70775.1 | 96 | 3,00E-108 | 40 |
|  | *Bipolaris sorokiniana* ND90Pr | XP_007700872.1 | 93 | 4,00E-107 | 44 |
|  | *Talaromyces stipitatus* ATCC 10500 | XP_002340035.1 | 95 | 6,00E-107 | 40 |
|  | *Bipolaris oryzae* ATCC 44560 | XP_007693481.1 | 93 | 2,00E-106 | 43 |
|  | *Pyrenophora teres* f. *teres* 0-1 | XP_003297838.1 | 95 | 3,00E-106 | 45 |
|  | *Monascus pilosus* | AGN71623.1 | 96 | 8,00E-105 | 42 |
|  | *Bipolaris maydis* ATCC 48331 | XP_014078431.1 | 93 | 1,00E-104 | 43 |
|  | *Endocarpon pusillum* Z07020 | XP_007801365.1 | 90 | 8,00E-103 | 41 |
|  | *Pestalotiopsis fici* W106-1 | XP_007833981.1 | 95 | 1,00E-102 | 39 |
|  | *Aspergillus terreus* NIH2624 | XP_001212621.1 | 94 | 8,00E-99 | 41 |
|  |  |  |  |  |  |
| **SOR6/SorF** | *Trichoderma reesei* QM6a | ETS06293.1 | 100 | 0.0 | 100 |
|  | *Trichoderma parareesei* CBS125925 | g12732 | 100 | 0.0 | 100 |
|  | *Trichoderma longibrachiatum* ATCC 18648 | Trilo3:1398857 | 100 | 0.0 | 100 |
|  | *Trichoderma citrinoviride* | Trici4:60343 | 100 | 0.0 | 100 |
|  | *Penicillium nordicum* | KOS45608.1 | 94 | 0.0 | 76 |
|  | *Acremonium chrysogenum* ATCC 11550 | KFH44385.1 | 95 | 0.0 | 77 |
|  | *Penicillium rubens* Wisconsin 54-1255 | XP_002567556.1 | 96 | 0.0 | 76 |
|  | *Aspergillus clavatus* NRRL 1 | XP_001267929.1 | 93 | 0.0 | 76 |
|  | *Ustilaginoidea virens* | KDB13142.1 | 96 | 0.0 | 77 |
|  | *Aspergillus nidulans* FGSC A4 | XP_660874.1 | 95 | 0.0 | 74 |
|  | *Aspergillus fumigatus* Af293 | XP_748509.1 | 95 | 0.0 | 78 |
|  | *Talaromyces cellulolyticus* | GAM41974.1 | 95 | 0.0 | 74 |
|  | *Talaromyces stipitatus* ATCC 10500 | XP_002486699.1 | 95 | 0.0 | 71 |
|  | *Exophiala xenobiotica* | XP_013322437.1 | 95 | 0.0 | 70 |
|  | *Neosartorya fischeri* NRRL 181 | XP_001258841.1 | 95 | 0.0 | 76 |
|  | *Chaetomium globosum* CBS 148.51 | XP_001227514.1 | 100 | 0.0 | 68 |
|  | *Oidiodendron maius* Zn | KIN00608.1 | 95 | 0.0 | 63 |
|  | *Trichoderma virens* Gv29-8 | XP_013956238.1 | 95 | 0.0 | 63 |
|  | *Pseudogymnoascus destructans* 20631-21 | XP_012746702.1 | 84 | 0.0 | 73 |
|  | *Trichoderma atroviride* IMI 206040 | XP_013945126.1 | 95 | 0.0 | 61 |
|  | *Trichoderma harzianum* | KKP04950.1 | 96 | 0.0 | 61 |
|  |  |  |  |  |  |
| **SOR7** | *Trichoderma reesei* QM6a | ETS06289.1 | 100 | 0.0 | 100 |
|  | *Trichoderma parareesei* CBS125925 | g12741 | 100 | 0.0 | 100 |
|  | *Trichoderma longibrachiatum* ATCC 18648 | Trilo3:1371623 | 100 | 0.0 | 100 |
|  | *Trichoderma citrinoviride* | Trici4:1172273 | 100 | 0.0 | 100 |
|  | *Trichoderma virens* Gv29-8 | XP_013959009.1 | 98 | 0.0 | 81 |
|  | *Trichoderma atroviride* IMI 206040 | XP_013946053.1 | 98 | 0.0 | 77 |
|  | *Rosellinia necatrix* | GAP88295.1 | 98 | 8,00E-150 | 60 |
|  | *Pseudogymnoascus* sp. VKM F-4520 (FW-2644) | KFZ10058.1 | 98 | 2,00E-128 | 60 |
|  | *Aureobasidium pullulans* EXF-150 | KEQ78695.1 | 98 | 1,00E-124 | 59 |
|  | *Grosmannia clavigera* kw1407 | XP_014176150.1 | 97 | 3,00E-122 | 58 |
|  | *Stachybotrys chartarum* IBT 7711 | KEY73625.1 | 98 | 3,00E-115 | 55 |
|  | *Talaromyces stipitatus* ATCC 10500 | XP_002483145.1 | 97 | 8,00E-104 | 46 |
|  | *Trichoderma harzianum* | KKP04490.1 | 53 | 2,00E-99 | 80 |
|  | *Talaromyces cellulolyticus* | GAM37295.1 | 96 | 8,00E-98 | 48 |
|  | *Talaromyces* *marneffei* ATCC 18224 | XP_002150938.1 | 95 | 6,00E-95 | 47 |
|  | *Aspergillus terreus* NIH2624 | XP_001209607.1 | 96 | 3,00E-94 | 48 |
|  | *Penicillium brasilianum* | CEO60941.1 | 83 | 3,00E-76 | 50 |
|  | *Talaromyces marneffei* PM1 | KFX45271.1 | 78 | 5,00E-72 | 48 |
|  | *Penicillium rubens* Wisconsin 54-1255 | XP_002560457.1 | 96 | 4,00E-70 | 36 |
|  | *Talaromyces cellulolyticus* | GAM36187.1 | 95 | 3,00E-67 | 37 |
|  |  |  |  |  |  |
| **SOR8** | *Trichoderma reesei* QM6a | XP_006961562.1 | 100 | 0.0 | 100 |
|  | *Trichoderma parareesei* CBS125925 | g12729 | 100 | 0.0 | 100 |
|  | *Trichoderma longibrachiatum* ATCC 18648 | Trilo3:1389527 | 100 | 0.0 | 100 |
|  | *Trichoderma citrinoviride* | Trici4:1128361 | 100 | 0.0 | 100 |
|  | *Acremonium chrysogenum* ATCC 11550 | KFH44377.1 | 94 | 0.0 | 68 |
|  | *Ustilaginoidea virens* | KDB17667.1 | 94 | 0.0 | 61 |
|  | *Aureobasidium subglaciale* EXF-2481 | XP_013346160.1 | 95 | 0.0 | 49 |
|  | *Aureobasidium pullulans* EXF-150 | KEQ81095.1 | 97 | 0.0 | 49 |
|  | *Aspergillus clavatus* NRRL 1 | XP_001276774.1 | 97 | 1,00E-177 | 49 |
|  | *Baudoinia panamericana* UAMH 10762 | XP_007673771.1 | 96 | 2,00E-173 | 49 |
|  | *Penicillium roqueforti* FM164 | CDM29285.1 | 99 | 6,00E-173 | 46 |
|  | *Aspergillus terreus* NIH2624 | XP_001209796.1 | 97 | 2,00E-171 | 46 |
|  | *Aspergillus kawachii* IFO 4308 | GAA82768.1 | 94 | 1,00E-168 | 49 |
|  | *Colletotrichum higginsianum* | CCF33474.1 | 97 | 2,00E-167 | 49 |
|  | *Colletotrichum gloeosporioides* Cg-14 | EQB52432.1 | 96 | 3,00E-167 | 49 |
|  | *Aspergillus fumigatus* Af293 | XP_747715.1 | 93 | 2,00E-166 | 48 |
|  | *Neosartorya* *fischeri* NRRL 181 | XP_001257567.1 | 93 | 2,00E-165 | 48 |

* accession numbers for *T. parareesei, T. longibrachiatum,* and *T. citrinoviride* are the protein IDs used in the genome annotation.
